# Exercise interventions to improve sleep quality in university students: a systematic review and meta-analysis of randomized controlled trials

**DOI:** 10.3389/fpubh.2026.1871771

**Published:** 2026-07-21

**Authors:** Dongzhi Nanjie, Ran Huo, Ning Su, Zeshuai Wang, Jian Wei, Jie Duo

**Affiliations:** 1 School of Tibetan Medicine, Qinghai University, Xining, Qinghai, China; 2 School of Physical Education and Training, Capital University of Physical Education and Sports, Beijing, China; 3 Faculty of Health Sciences and Sports, Macao Polytechnic University, Macao, China; 4 School of Wushu, Shanghai University of Sport, Shanghai, China; 5 Department of Otolaryngology, Ningxia Hospital of Integrated Traditional Chinese and Western Medicine, Yinchuan, Ningxia, China

**Keywords:** exercise, meta-analysis, physical activity, Pittsburgh Sleep Quality Index, randomized controlled trial, sleep quality, university students

## Abstract

**Background and objective:**

Poor sleep quality is common among university students and is associated with adverse academic, psychological, and cardiometabolic outcomes. Exercise may provide a scalable, low-cost, non-pharmacological approach to improving sleep. Although previous reviews have examined physical activity and sleep in university students, evidence specifically derived from randomized controlled trials of structured exercise interventions using the Pittsburgh Sleep Quality Index (PSQI) has not been adequately synthesized. This systematic review and meta-analysis therefore evaluated the effects of structured exercise interventions on the PSQI global score and PSQI-derived component scores in university students.

**Methods:**

We searched PubMed, Web of Science, Embase, CENTRAL, CINAHL, PsycINFO, and ProQuest Dissertations & Theses from inception to April 30, 2026. Randomized controlled trials comparing structured exercise interventions with non-exercise controls in university students and reporting PSQI-based outcomes were eligible. The primary outcome was the PSQI global score. Secondary outcomes were the PSQI component scores for sleep latency, sleep duration, habitual sleep efficiency, and sleep disturbances. Mean differences (MDs) with 95% confidence intervals (CIs) were pooled using DerSimonian–Laird random-effects models. Heterogeneity was assessed using I^2^ and τ^2^. Robustness was examined using leave-one-out analyses, change-from-baseline analyses, prediction intervals, restricted maximum-likelihood estimation, and the Hartung–Knapp adjustment. Certainty of evidence was assessed using GRADE.

**Results:**

Seven randomized controlled trials involving 701 participants contributed eight exercise-vs.-control comparisons. Six trials contributing seven comparisons and 602 participants were included in the primary meta-analysis of the PSQI global score. Exercise reduced the PSQI global score compared with non-exercise controls (MD = −2.43, 95% CI −3.72 to −1.14; *p* < 0.001; *I*^2^ = 91.7%). This finding remained statistically significant in leave-one-out and change-from-baseline sensitivity analyses, as well as under restricted maximum-likelihood estimation (MD = −2.43, 95% CI −3.68 to −1.18) and the Hartung–Knapp adjustment (MD = −2.43, 95% CI −3.98 to −0.88; *p* = 0.009). However, the 95% prediction interval ranged from −6.98 to 2.12 and included the null value, indicating substantial variation in the likely effects across settings. Three trials contributing three comparisons and 290 participants were included in each analysis of the PSQI-derived component scores. Exercise reduced the PSQI sleep-duration component score (MD = −0.74, 95% CI −1.02 to −0.46; *I*^2^ = 56.9%) and the PSQI habitual sleep-efficiency component score (MD = −0.43, 95% CI −0.64 to −0.22; *I*^2^ = 44.9%). Effects on the PSQI sleep-latency component score (MD = −0.53, 95% CI −1.36 to 0.30; *I*^2^ = 96.2%) and the PSQI sleep-disturbance component score (MD = −0.54, 95% CI −1.23 to 0.15; *I*^2^ = 94.5%) favored exercise but were not statistically significant. Certainty of evidence was very low for the PSQI global score and the sleep-duration, sleep-latency, and sleep-disturbance component scores, and low for the habitual sleep-efficiency component score.

**Conclusion:**

Structured exercise interventions may improve overall sleep quality in university students, with the most consistent benefits observed for the PSQI sleep-duration and habitual sleep-efficiency component scores. However, substantial heterogeneity, a prediction interval that included no effect, and very low to low certainty of evidence limit confidence in the pooled findings. Larger, rigorously designed randomized trials using standardized intervention protocols and both subjective and objective sleep measures are needed before definitive practice recommendations can be made.

**Systematic review registration:**

www.crd.york.ac.uk/prospero, identifier: CRD420261384510.

## Introduction

Sleep is increasingly recognized as a fundamental determinant of physical, mental, and cognitive health, rather than a passive state of rest. Healthy sleep encompasses not only adequate duration but also good subjective quality, appropriate timing, regularity, and the absence of clinically significant sleep disorders (1, 2). Disturbances in sleep duration, continuity, and circadian alignment have been linked to impaired emotional regulation, reduced executive function, metabolic dysregulation, and increased cardiometabolic risk (1, 3). Among sleep-related complaints, poor sleep quality and insomnia symptoms—characterized by persistent difficulties in initiating or maintaining sleep, early-morning awakening, or non-restorative sleep despite adequate opportunity, accompanied by daytime impairment such as fatigue, reduced concentration, and emotional disturbance (4)—are particularly consequential for populations whose daily functioning depends heavily on cognitive performance and emotional resilience (5–7).

University students represent one such population, with a disproportionately high burden of poor sleep. The transition from adolescence to early adulthood is accompanied by major changes in living environment, academic demands, social routines, lifestyle autonomy, and digital media exposure, all of which may destabilize sleep–wake regulation (5, 6, 8). A recent global meta-analysis estimated the pooled prevalence of insomnia symptoms among undergraduate students at approximately 47% (9), substantially exceeding estimates reported in the general adult population. This vulnerability appears to be driven by interacting mechanisms: evening use of electronic devices and social media can delay sleep onset through blue-light exposure, cognitive arousal, and the displacement of sleep opportunity (10, 11), while academic stress, anxiety, depressive symptoms, and ruminative thinking sustain a state of cognitive and physiological hyperarousal that disrupts the normal transition from wakefulness to sleep (8, 12, 13). Within a public-health framework, poor sleep among university students should therefore be viewed not as a transient lifestyle inconvenience, but as a modifiable population-level health concern with implications for academic achievement, mental wellbeing, and long-term cardiometabolic and psychiatric outcomes (3, 5–7).

Evidence-based treatments for chronic insomnia emphasize behavioral and psychological interventions—most notably cognitive behavioral therapy for insomnia (CBT-I)—as first-line care (14, 15). Pharmacological treatment may be considered in selected cases, but its use is limited by concerns regarding residual daytime sedation, impaired cognitive performance, tolerance, dependence, and its failure to address the behavioral and psychological drivers of insomnia (16, 17). These limitations are especially salient for university students, whose academic responsibilities demand sustained attention, memory consolidation, and daytime alertness (6, 7). Although CBT-I demonstrates strong efficacy, its real-world implementation in campus settings is constrained by limited access to trained providers, high time demands, low adherence to behavioral protocols, and a poor fit with irregular student schedules and shared living environments (18–21). From a public-health perspective, there is therefore a pressing need for scalable, acceptable, low-risk, and non-pharmacological strategies that can be embedded into students' daily routines.

Exercise interventions offer a biologically plausible and practically feasible approach to improving sleep quality in university students. Regular physical activity may enhance sleep through multiple converging pathways, including thermoregulatory changes that facilitate sleep onset, modulation of circadian rhythms and melatonin secretion (22–24), downregulation of hypothalamic–pituitary–adrenal axis hyperactivity (25, 26), reduction of systemic inflammation, and psychological benefits such as improved mood and self-efficacy and reduced ruminative thinking (27–29). Different modalities—including aerobic exercise, resistance training, and mind–body practices such as yoga and Tai Chi—may exert partly distinct effects on sleep initiation, continuity, efficiency, and perceived quality (24, 30, 31). However, the available evidence remains heterogeneous with respect to exercise type, intensity, duration, frequency, outcome measurement, and methodological quality (32, 33).

Although a previous systematic review examined physical activity and sleep among university students (34), it addressed a broader evidence base rather than specifically synthesizing randomized controlled trials of structured exercise interventions. Consequently, the effects attributable to structured exercise, particularly on the PSQI global score and its individual component scores, remain insufficiently quantified. Other systematic reviews of exercise and sleep have predominantly focused on general adult, older, or clinical populations (32, 33), and the extent to which their findings generalize to university students—a population with distinctive developmental, academic, and behavioral characteristics—remains uncertain. A trial-only synthesis is therefore needed to clarify the effects of structured exercise on overall sleep quality and specific dimensions of sleep in this population.

To address this more precisely defined evidence gap, we conducted a systematic review and meta-analysis of randomized controlled trials evaluating structured exercise interventions, compared with non-exercise control conditions, in university students. The primary quantitative outcome was overall sleep quality, assessed using the Pittsburgh Sleep Quality Index (PSQI) global score. Secondary quantitative outcomes included the PSQI component scores for sleep latency, sleep duration, habitual sleep efficiency, and sleep disturbances. Eligible sleep outcomes assessed using other validated instruments were retained in the systematic review but were summarized narratively when they could not be directly harmonized with PSQI-based outcomes. We also explored whether intervention effects differed according to participant and intervention characteristics and considered the implications of the findings for campus-based sleep-health strategies.

## Methods

### Protocol registration and reporting guidelines

This systematic review and meta-analysis was conducted and reported in accordance with the Preferred Reporting Items for Systematic Reviews and Meta-Analyses (PRISMA) 2020 statement (35). The protocol was prospectively registered in the International Prospective Register of Systematic Reviews (PROSPERO; registration number: CRD420261384510).

### Search strategy

A comprehensive literature search was independently performed by two reviewers in seven electronic databases: PubMed, Web of Science, Embase, the Cochrane Central Register of Controlled Trials (CENTRAL), CINAHL, PsycINFO, and ProQuest Dissertations & Theses. Databases were searched from inception to April 30, 2026. The search strategy was developed based on the PICOS framework (36) and adapted for each database using controlled vocabulary terms and free-text terms.

The search strategy combined three groups of terms related to the population, intervention, and outcome. Population terms included “university students,” “college students,” and “undergraduates”; intervention terms included “exercise,” “physical activity,” “aerobic exercise,” “resistance training,” “yoga,” “Tai Chi,” “Pilates,” and “mind–body exercise”; and outcome terms included “sleep quality,” “Pittsburgh Sleep Quality Index,” “PSQI,” and “sleep.” Medical Subject Headings (MeSH) and free-text terms were combined using Boolean operators. The search was supplemented by manually screening the reference lists of included studies and relevant systematic reviews to identify additional eligible trials. No restrictions were imposed on publication language, intervention duration, or exercise modality. The full search strategies for each database are presented in Supplementary Table S1.

### Eligibility criteria

Eligibility criteria were defined a priori according to the PICOS framework (36). Studies were eligible for inclusion if they met all of the following criteria: participants were university or college students, with no restriction on age, sex, ethnicity, or baseline sleep status; the intervention consisted of a structured exercise program, including but not limited to aerobic exercise, resistance training, mind–body exercise such as yoga, Tai Chi, or Qigong, or Pilates; the comparator was a non-exercise control, including usual care, no intervention, health education, wait-list control, or attention control; sleep quality was assessed using the Pittsburgh Sleep Quality Index (PSQI) (4) or another validated self-reported or objective sleep-quality measure; and the study was designed as a randomized controlled trial.

Studies were excluded if they were non-randomized or quasi-experimental studies, observational studies, narrative or systematic reviews, animal studies, conference abstracts or protocols without sufficient outcome data, or trials in which exercise was not the primary or independently evaluable intervention component, such as multimodal lifestyle programs in which the independent effect of exercise could not be isolated.

### Study selection

All retrieved records were imported into EndNote 21 (Clarivate Analytics, Philadelphia, PA, USA), and duplicates were removed. Two reviewers independently screened the titles and abstracts of all unique records against the eligibility criteria. The full texts of potentially relevant articles were then retrieved and independently assessed for inclusion. Disagreements at either stage were resolved through discussion, and any remaining discrepancies were adjudicated by a third reviewer. Inter-reviewer agreement was monitored throughout the screening process by comparing independent judgements and quantifying the proportion of records on which the reviewers disagreed.

### Data extraction

Two reviewers independently extracted data from each eligible study using a standardized data extraction form. Extracted information included publication details, country, study design, participant characteristics, sample size, intervention characteristics, comparator type, intervention duration, exercise frequency, session length, exercise intensity, supervision status, outcome measures, assessment time points, and post-intervention means, standard deviations, and sample sizes for each outcome. Any discrepancies were resolved through discussion or consultation with a third reviewer.

For quantitative synthesis, post-intervention values were preferentially extracted for all outcomes. When standard errors, confidence intervals, *p-values*, or other summary statistics were reported instead of standard deviations, standard deviations were calculated according to standard methods recommended in the Cochrane Handbook for Systematic Reviews of Interventions (37). When essential data were missing or unclear, the corresponding authors were contacted via email for clarification; studies were excluded from the quantitative synthesis when sufficient numerical data could not be obtained.

For multi-arm trials, data were handled in accordance with Cochrane guidance (37) to avoid double-counting of participants. In the trial by Iyer et al. (59), multiple eligible exercise intervention arms were combined into a single exercise group for the primary analysis, with the combined mean and pooled standard deviation calculated using standard formulae from the Cochrane Handbook (37). In the trial by Zhou et al. (39), two eligible exercise intervention arms were retained as separate comparisons because they represented distinct exercise modalities; to avoid double-counting the shared control group, the control sample size was divided equally between the two relevant comparisons, while the control group mean and standard deviation were kept unchanged.

### Risk of bias assessment

The risk of bias of included randomized controlled trials was independently assessed by two reviewers using the revised Cochrane Risk of Bias tool for randomized trials (RoB 2) (40). Five domains were evaluated: bias arising from the randomization process, bias due to deviations from intended interventions, bias due to missing outcome data, bias in measurement of the outcome, and bias in selection of the reported result. Because all included trials assessed sleep quality through self-reported instruments and exercise interventions could not be blinded to participants, the domain concerning measurement of the outcome was assessed with particular care, taking into account whether the absence of blinding was likely to have substantively biased outcome assessment. Each domain, as well as the overall risk of bias, was judged as “low risk of bias,” “some concerns,” or “high risk of bias” according to the algorithms specified in the RoB 2 guidance (40). Disagreements were resolved through discussion, and unresolved discrepancies were adjudicated by a third reviewer. Risk of bias judgments was visualized using the robvis tool (41).

### Certainty of evidence

The certainty of evidence for each outcome was assessed using the Grading of Recommendations Assessment, Development and Evaluation (GRADE) approach (42). Evidence from randomized controlled trials was initially rated as high certainty and could be downgraded by one or two levels for risk of bias, inconsistency, indirectness, imprecision, or publication bias (43–47). The overall certainty of evidence for each outcome was classified as high, moderate, low, or very low, and summarized in a GRADE Summary of Findings table generated using GRADEpro GDT software (48).

### Statistical analysis

All statistical analyses were performed using Stata (version 18.0; StataCorp, College Station, TX, USA), with the primary meta-analytic procedures implemented through the user-written metan package (49) and supplementary robustness analyses conducted using Stata's official meta commands. The primary outcome was global sleep quality, assessed using the Pittsburgh Sleep Quality Index (PSQI) global score (4), a widely validated composite measure of overall sleep quality in which higher scores reflect poorer sleep. Secondary outcomes comprised four PSQI-derived sleep parameters that were consistently reported across the included trials: sleep disturbances, sleep duration, habitual sleep efficiency, and sleep latency; the remaining PSQI components were not pooled because of inconsistent reporting across studies. Quantitative syntheses were restricted to studies reporting the same PSQI global or component score. Non-PSQI outcomes that could not be directly harmonized with the corresponding PSQI scale were summarized narratively. Accordingly, the nightly sleep-duration data from de Vries et al., which were reported in hours rather than as a PSQI component score, were excluded from the PSQI-specific quantitative analysis. Specifically, de Vries et al. assessed sleep duration using a single self-report item measuring mean nightly sleep hours, in which higher values indicate longer sleep duration — a direction opposite to that of the PSQI sleep duration component score (range 0–3), in which higher scores reflect shorter and poorer-quality sleep. Because the two measures differ in both scale and scoring direction, direct harmonization was not feasible, and the data from de Vries et al. were therefore summarized narratively alongside the quantitative synthesis of PSQI-based sleep duration outcomes. For each randomized controlled trial, post-intervention means, standard deviations, and sample sizes were extracted independently for the exercise intervention and control arms.

Because the studies included in each quantitative synthesis used the same PSQI scale, pooled treatment effects were expressed as mean differences (MDs) with 95% confidence intervals (CIs) rather than standardized mean differences, in order to preserve the clinical interpretability of the effect estimates on the original measurement scale (37). For all outcomes—including the PSQI global score and the four PSQI component scores—higher scores indicated poorer sleep, and a negative MD therefore indicated a benefit favoring the exercise group. Given the anticipated clinical and methodological heterogeneity across studies—arising from differences in exercise modality, intervention duration, frequency, intensity, level of supervision, comparator conditions, and participant characteristics—a random-effects model based on the DerSimonian–Laird method (50) was selected a priori for all primary analyses. Statistical heterogeneity was assessed using Cochran's Q test and quantified with the I^2^ and τ^2^ statistics (51), with values of approximately 25%, 50%, 75%, and >75% interpreted as low, moderate, substantial, and considerable heterogeneity, respectively, in accordance with the Cochrane Handbook for Systematic Reviews of Interventions (37).

The robustness of the pooled estimate for the primary outcome was examined through leave-one-out sensitivity analysis (52), in which each study was sequentially omitted and the pooled effect size recalculated to identify potentially influential trials. To examine the potential influence of baseline differences across trials, an additional sensitivity analysis was performed using change-from-baseline scores. When change-score standard deviations were not reported, they were estimated from baseline and post-intervention standard deviations assuming a pre–post correlation coefficient of (*r* = 0.5), in accordance with Cochrane guidance. The reported change-score standard deviations were used directly for Li et al. (58). These analyses were conducted for the PSQI global score and component outcomes when sufficient data were available, with full results presented in Supplementary Appendix 11.

Given the small number of comparisons and considerable heterogeneity in the primary analysis, three additional robustness analyses were performed for the PSQI global score. First, a 95% prediction interval was calculated under the original DerSimonian–Laird model. Second, restricted maximum-likelihood estimation was applied as an alternative estimator of the between-study variance, together with a corresponding prediction interval. Third, the analysis was repeated using REML estimation with the Hartung–Knapp adjustment and a 95% prediction interval. These analyses were also applied to secondary outcomes when sufficient directly comparable data were available. Full results are presented in Supplementary Appendix 12.

Subgroup analyses were planned according to sex composition and intervention duration to explore whether intervention effects on sleep quality differed across these strata. An additional *post hoc* exploratory subgroup analysis by exercise modality was conducted for the PSQI global score only. Because most exercise modalities were represented by a single study or comparison, this analysis was undertaken descriptively to explore potential sources of heterogeneity rather than to provide definitive between-modality comparisons. Exercise-modality subgroup analyses were not performed for the secondary outcomes because only three trials contributed data to these analyses; given the limited number of studies within certain subgroups, these subgroup findings were considered exploratory and hypothesis-generating rather than confirmatory. Parallel sensitivity and subgroup analyses for the secondary outcomes were performed where the number of contributing studies permitted. Potential small-study effects and publication bias for the primary outcome were assessed through visual inspection of funnel plots, and Egger's regression test (53) was performed as an exploratory analysis only, as formal tests of funnel plot asymmetry have limited statistical power when fewer than 10 studies are pooled (54); accordingly, publication bias was interpreted with caution and was not judged solely on the basis of Egger's test. Egger's test was not performed for outcomes with too few studies to provide an estimable result.

All statistical tests were two-sided, and a *p-value* < 0.05 was considered statistically significant. The interpretation of findings was based not on statistical significance alone, but on the magnitude and precision of the pooled effect estimates, the degree of between-study heterogeneity, the prediction intervals, and the consistency of results across sensitivity and subgroup analyses, in line with current methodological guidance for systematic reviews and meta-analyses (37).

## Results

### Study identification and selection

**Figure 1:**
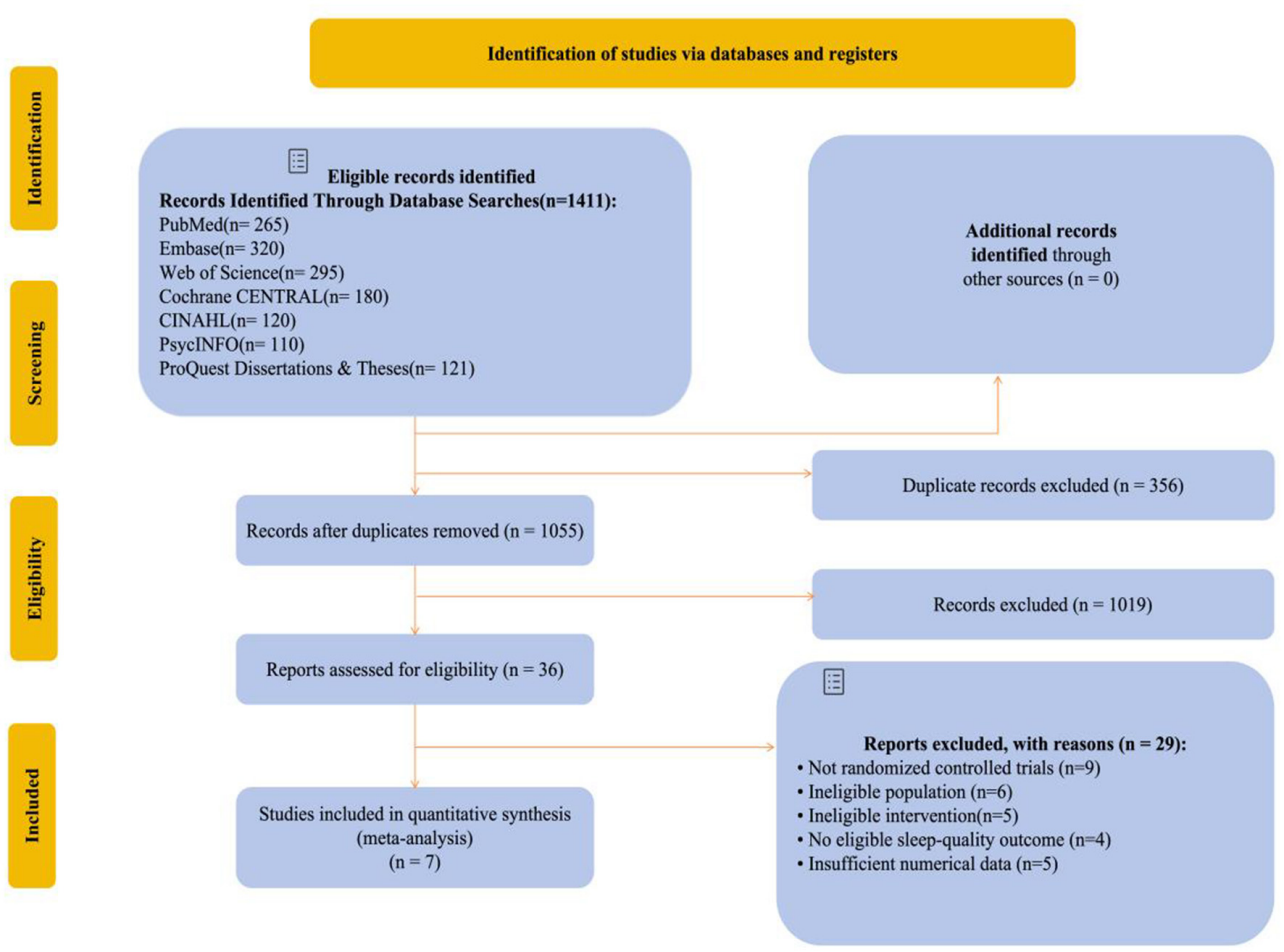
Flow chart of the search for eligible RCTs.

The database searches identified 1,411 records. After removal of 356 duplicates, 1,055 unique records were screened by title and abstract, of which 1,019 were excluded as irrelevant. The full texts of 36 potentially eligible reports were retrieved and assessed for eligibility. Of these, 29 reports were excluded for the reasons shown in Figure 1, most commonly because they used a non-randomized design, enrolled an ineligible population, did not evaluate an exercise-based intervention, did not report eligible sleep-quality outcomes, or provided insufficient numerical data. Manual screening of reference lists and relevant reviews did not identify additional eligible trials. Disagreements between the two reviewers occurred in fewer than 5% of records during both title/abstract and full-text screening; all disagreements were resolved through discussion or, where necessary, by adjudication of a third reviewer. Ultimately, seven randomized controlled trials were included in the systematic review and meta-analysis. The study identification and selection process is presented in the PRISMA 2020 flow diagram (Figure 1).

### Study characteristics

Seven randomized controlled trials were included in the systematic review (38, 39, 55–59), of which six contributed data to the PSQI-based meta-analysis; detailed characteristics are presented in Table 1. Because one trial (39) included two distinct exercise modalities that were retained as separate comparisons, the seven trials represented a total of eight exercise-vs.-control comparisons across the review. The primary meta-analysis of the PSQI global score included six trials contributing seven pairwise comparisons and 602 participants (335 in the exercise groups and 267 in the control groups). The included studies were published between 2015 and 2026 and involved 701 participants in total, with sample sizes ranging from 35 to 206 per trial. All studies used a parallel-group randomized controlled trial design; two trials were three-arm trials (39, 59), in which the intervention arms were handled according to the predefined meta-analytic approach to avoid double-counting of participants. In Iyer et al. (59), the two yoga intervention arms were combined into a single exercise group, whereas in Zhou et al. (39), the shared control group was divided equally between the Tai Chi and brisk-walking comparisons for the meta-analysis.

**Table 1:** Characteristics of included studies.

References	Country	Design	Sample size (E/C)	Age (years), mean ± SD	Female (%) (E/C)	BMI (kg/m^2^), mean ± SD	Baseline PSQI global score, mean ± SD	Intervention	Comparator	Duration (weeks)	Frequency ( × /week)	Session (min)	Intensity	Supervision	Outcomes
Fu et al. (55)^a^	China	RCT	17/18	19.2 ± 1.1/19.3 ± 1.3	100/100	21.8 ± 1.0/22.1 ± 2.0	5.1 ± 3.4/4.8 ± 2.5	HIIT	Non-exercise	4	5	34 ^a^	>90% VO_2_max	Supervised	PSQI
Ezati et al. (56)	Iran	RCT	32/35	20.5 ± 1.6/20.1 ± 1.3	100/100	21.8 ± 2.1/22.8 ± 2.3	6.5 ± 2.4/6.3 ± 2.6	Aerobic exercise	Non-exercise	8	3	60	45–70% HRmax	Supervised	PSQI, SL, SDur, SE, SDis
Amzajerdi et al. (57)	Iran	RCT	32/35	20.8 ± 1.3/20.1 ± 1.3	100/100	22.1 ± 2.0/22.8 ± 2.3	6.3 ± 2.7/6.3 ± 2.6	Pilates	Non-exercise	8	3	60	NR	Supervised	PSQI, SL, SDur, SE, SDis
Li et al. (58)	China	RCT	101/105	20.6 ± 1.0/20.9 ± 1.2	85.1/80.0	NR	3.7 ± 1.5/4.1 ± 1.7	Tai Chi (Baduanjin)	Non-exercise	12	5	60	3.0–6.0 METs	Supervised	PSQI
de Vries et al. (38)^b^	Netherlands	RCT	50/49	20.9 ± 2.5/20.7 ± 2.2	82.0/79.6	NR	—	Aerobic exercise	Non-exercise	6	3	60	Low	Partly supervised	—
Iyer et al. (59)^c^	India	RCT	104/52	22.2 ± 1.9/22.3 ± 1.6	41.3/48.1	23.0 ± 4.3/22.6 ± 5.1	9.2 ± 4.0/9.3 ± 3.4	Yoga	Non-exercise	4	5	60	Mild	Online supervised	PSQI, SL, SDur, SE, SDis
Zhou et al. (39)^d^ (Tai Chi)	China	RCT	25/22	19.2 ± 0.7/18.9 ± 0.6	84.0/68.2	21.4 ± 2.3/20.7 ± 3.0	7.2 ± 1.6/7.0 ± 1.1	Tai Chi	Non-exercise	24	3	60	Light–moderate	Supervised	PSQI
Zhou et al. (39)^d^ (Brisk walking)	China	RCT	24/22	19.0 ± 0.7/18.9 ± 0.6	70.8/68.2	21.4 ± 3.3/20.7 ± 3.0	6.9 ± 1.7/7.0 ± 1.1	Aerobic exercise (brisk walking)	Non-exercise	24	3	60	60–80% HRmax	Supervised	PSQI

BMI, body mass index; C, control group; E, exercise group; HIIT, high-intensity interval training; HRmax, maximum heart rate; METs, metabolic equivalents; NR, not reported; PSQI, Pittsburgh Sleep Quality Index; RCT, randomized controlled trial; SD, standard deviation; SDis, sleep disturbances; SDur, sleep duration; SE, sleep efficiency; SL, sleep latency; VO_2_max, maximal oxygen uptake.

^a^Fu (2025): each HIIT session included a 10-min warm-up, 9 min of high-intensity intervals, and 15 min of stretching/relaxation.

^b^de Vries et al. (38) assessed sleep duration as self-reported mean nightly hours (higher values = longer sleep), which is opposite in scoring direction to the PSQI sleep-duration component score (higher scores = poorer sleep) and was therefore excluded from quantitative synthesis and summarized narratively.

^c^Iyer et al. (59): the two yoga arms were combined for the primary analysis.

^d^Zhou et al. (39): Tai Chi and brisk walking arms were retained as separate comparisons; the shared control group was split to avoid double-counting.

The mean age of participants ranged from 18.9 to 22.3 years. Three trials enrolled female participants exclusively (55–57), whereas the remaining four included mixed-sex samples; among the mixed-sex trials, females accounted for 69.3% and males for 30.7% of participants. Three trials were conducted in China (39, 55, 58), two in Iran (56, 57), one in India (59), and one in the Netherlands (38).

Baseline sleep quality differed notably across trials, with mean PSQI global scores ranging from 3.7 to 9.3 among the six trials that administered the PSQI; de Vries et al. (38) used a non-PSQI sleep measure and was summarized narratively. Exercise modalities included aerobic exercise, high-intensity interval training, Tai Chi, yoga, Pilates, and brisk walking. Intervention duration ranged from 4 to 24 weeks (median, 8 weeks), with sessions delivered three to five times per week and lasting approximately 34 to 60 min per session. Most interventions were supervised by qualified instructors, exercise specialists, or coaches.

### Risk of bias of included studies

Among the seven included randomized controlled trials (eight independent comparisons), one trial was judged to be at overall low risk of bias (58), whereas the remaining six were rated as having some concerns (38, 39, 55–59). No study was judged to be at high risk of bias. All trials were assessed as low risk in the domain of missing outcome data (D3). With respect to measurement of the outcome (D4), six trials were rated as having some concerns because sleep quality was assessed using the self-reported PSQI, and participants could not be blinded to their exercise allocation; awareness of group assignment may plausibly have influenced self-reported responses. Li et al. (58) was the sole exception, rated as low risk in D4, as the original report explicitly stated that outcome assessors were blinded to treatment allocation.

**Figure 2:**
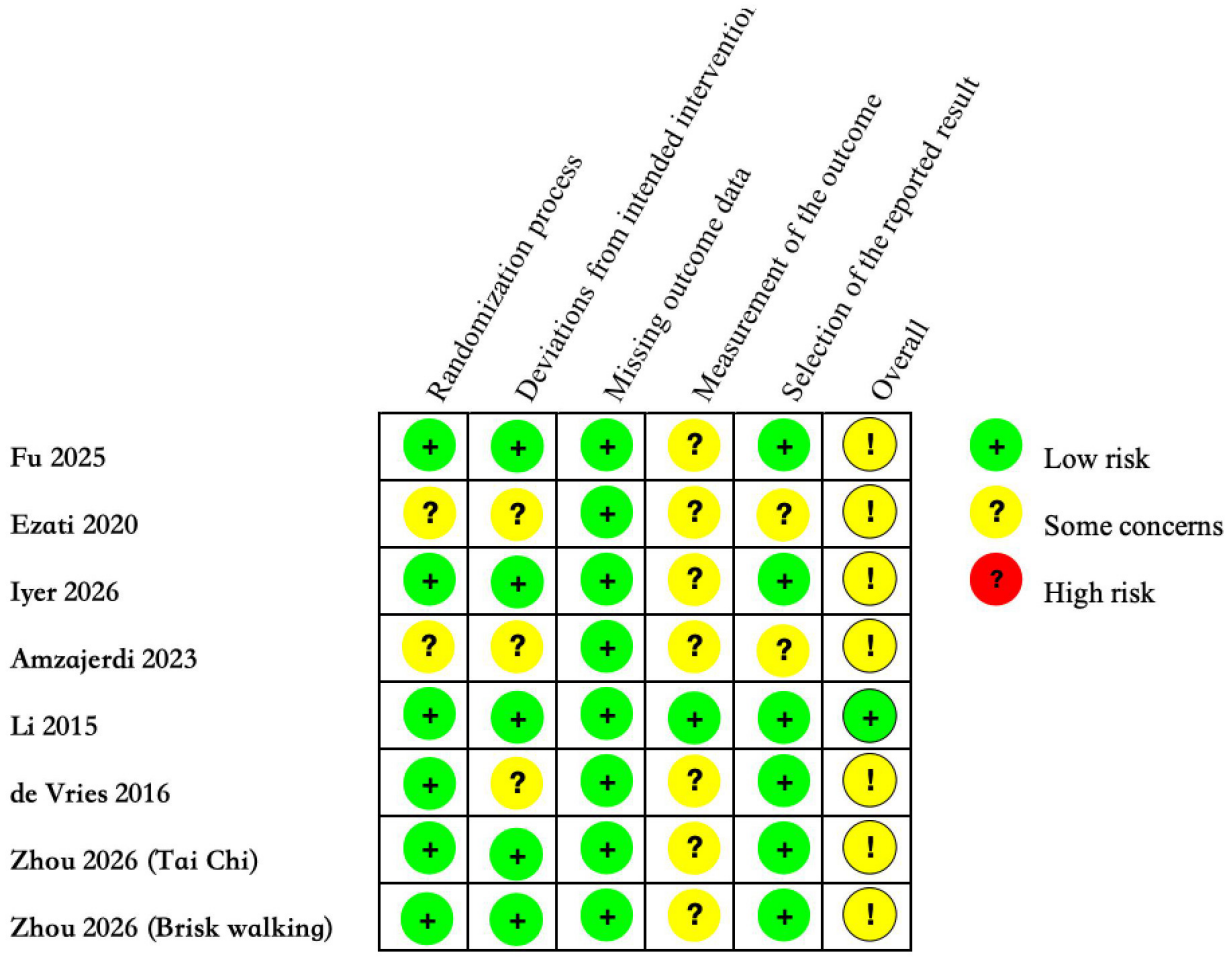
Assessment of risk of bias.

The main sources of concern arose from the randomization process (D1) and deviations from intended interventions (D2). Two trials were rated as having some concerns in the randomization process domain (56, 57), reflecting limited reporting of random sequence generation and allocation concealment. Three trials were rated as having some concerns in the deviations-from-intended-interventions domain (38, 56, 57); for the trial by de Vries et al. (38), this judgement was driven by the partially unsupervised nature of the intervention, in which one of the three weekly sessions was completed independently by participants and adherence was self-reported. Concerns regarding selection of the reported result (D5) were identified in two trials (56, 57), reflecting insufficient information on prespecified analysis plans or prospective trial registration. Overall, only Li et al. (58) was judged to be at low risk of bias across all five domains; the remaining six trials were rated as having some concerns, driven primarily by limitations in outcome measurement blinding, trial conduct, and prespecified analysis plans. Detailed domain-level assessments are shown in Figure 2.

### Effects on PSQI global score

**Figure 3:**
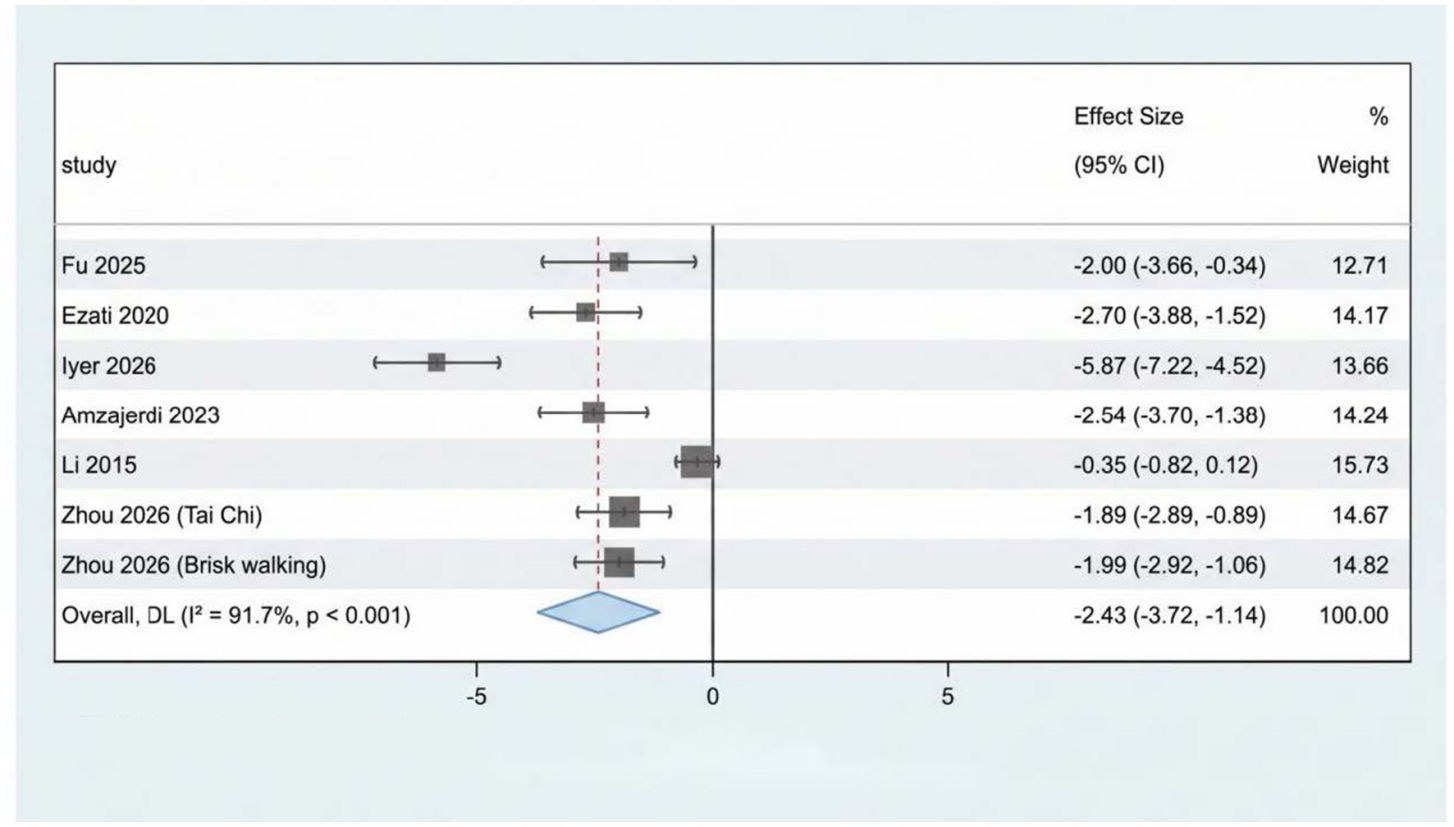
Forest plot of the effect of exercise interventions on PSQI global score. Weights are from random-effects model.

Six trials providing seven independent comparisons (*n* = 602 participants) contributed to the primary meta-analysis of the PSQI global score (Figure 3). Compared with non-exercise controls, exercise interventions resulted in a statistically significant reduction in post-intervention PSQI global score, indicating an improvement in overall sleep quality (pooled MD = −2.43, 95% CI −3.72 to −1.14; *p* < 0.001). The direction of effect favored exercise in all comparisons, although the confidence interval crossed the line of no effect for the trial by Li et al. (58), in which the effect estimate was small (MD = −0.35, 95% CI −0.82 to 0.12). The largest treatment effect was observed in the trial by Iyer et al. (59), in which yoga was delivered to participants with clinically elevated baseline PSQI scores (MD = −5.87, 95% CI −7.22 to −4.52).

Between-study heterogeneity was considerable (Cochran's *Q* = 72.20, *df* = 6, *p* < 0.001; *I*^2^ = 91.7%, 95% CI 35.9% to 97.0%; τ^2^ = 2.70), reflecting substantial variability in the magnitude of effect estimates across trials. This variability appeared to be related, at least in part, to differences in baseline sleep status, intervention modality, and intervention duration, which were further explored in subgroup and sensitivity analyses described below. The 95% prediction interval for the pooled estimate (−6.98 to 2.12) crossed the line of no effect, indicating that while the average effect favored exercise, the true effect in a new comparable setting may range from a substantial improvement to no benefit, and should be interpreted with caution.

### Effects on PSQI-derived sleep parameters

**Figure 4:**
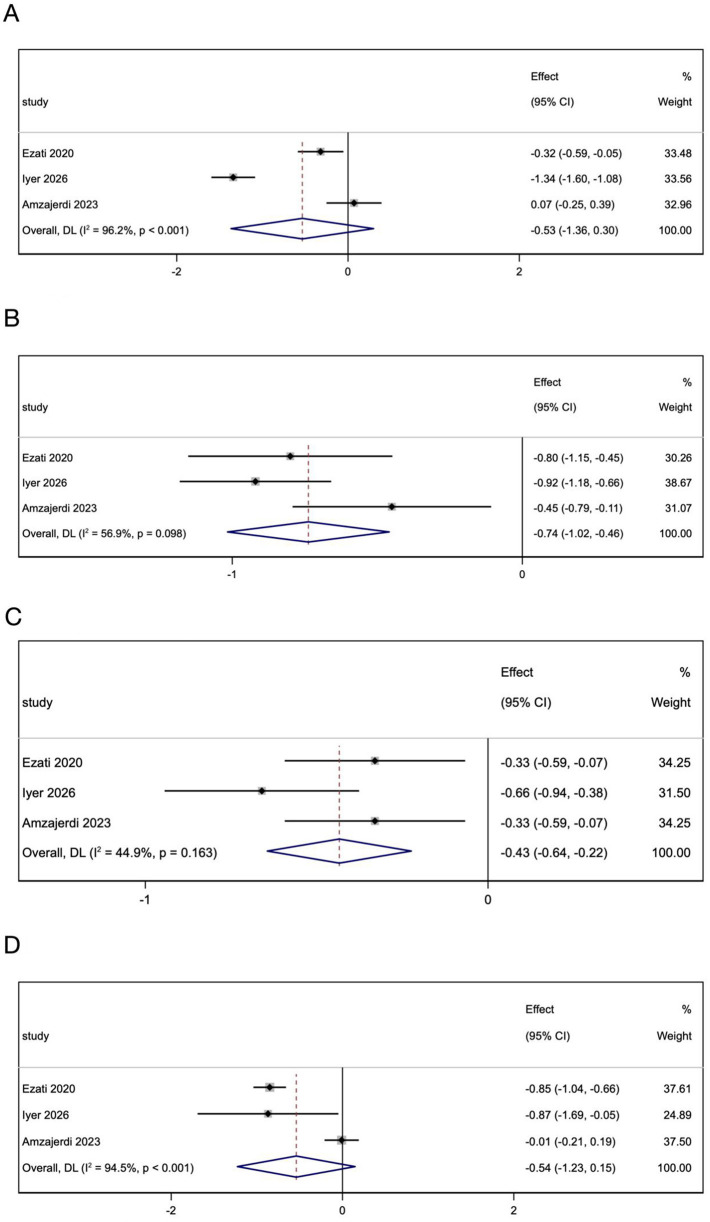
Forest plots of the effects of exercise interventions on PSQI-derived sleep parameters. **(A)** Sleep latency. **(B)** Sleep duration. **(C)** Sleep efficiency. **(D)** Sleep disturbances. Weights are from random-effects model.

Secondary analyses examined four PSQI-derived component scores: sleep disturbances, sleep duration, sleep efficiency, and sleep latency. For all components, higher scores reflect poorer sleep quality. Three trials contributing three comparisons and 290 participants reported data for all four PSQI component outcomes (Figures 4A–D).

Exercise interventions produced statistically significant improvements in sleep duration scores (3 trials; MD = −0.74, 95% CI −1.02 to −0.46; *p* < 0.001; *I*^2^ = 56.9%) and sleep efficiency scores (3 trials; MD = −0.43, 95% CI −0.64 to −0.22; *p* < 0.001; *I*^2^ = 44.9%), indicating that exercise was associated with more adequate sleep duration and improved sleep efficiency. For sleep latency (3 trials; MD = −0.53, 95% CI −1.36 to 0.30; *p* = 0.207; *I*^2^ = 96.2%) and sleep disturbances (3 trials; MD = −0.54, 95% CI −1.23 to 0.15; *p* = 0.128; *I*^2^ = 94.5%), pooled estimates consistently favored exercise but did not attain statistical significance. The nightly sleep-duration data from de Vries et al. were not included in this analysis, as that study assessed sleep duration in hours rather than using the PSQI sleep-duration component score; its findings are summarized narratively in Supplementary Appendix 4. Detailed forest plots for all PSQI-derived parameters are presented in Supplementary Appendix Figures S4.2–S4.5.

Heterogeneity was substantial for sleep latency (*I*^2^ = 96.2%) and sleep disturbances (*I*^2^ = 94.5%), limiting the interpretability of these pooled estimates. Heterogeneity was moderate for the PSQI sleep-duration component score (*I*^2^ = 56.9%) and low to moderate for the PSQI habitual sleep-efficiency component score (*I*^2^ = 44.9%). Sensitivity analyses for these outcomes are presented in Supplementary Appendix Figures S5.2–S5.5.

### Sensitivity and subgroup analyses

A leave-one-out sensitivity analysis was conducted for the primary outcome (PSQI global score) by sequentially omitting each comparison under a random-effects model. The pooled estimate remained statistically significant across all iterations, consistently favoring exercise, with mean differences ranging from −2.80 to −1.85 and all 95% confidence intervals excluding zero (Supplementary Appendix Figure S5.1). The pooled effect was most sensitive to exclusion of the trial by Iyer et al., which reduced the magnitude of the effect (MD = −1.85, 95% CI −2.80 to −0.89), reflecting its relatively large treatment effect. In contrast, exclusion of the trial by Li et al. yielded the largest pooled estimate (MD = −2.80, 95% CI −3.91 to −1.70), suggesting a potential attenuating influence of this study. Importantly, no single study materially altered the direction or statistical significance of the overall effect.

Sensitivity analyses for secondary sleep outcomes demonstrated similar robustness, with no individual study exerting a disproportionate influence on the pooled estimates (Supplementary Appendix Figures S5.2–S5.5).

A change-from-baseline sensitivity analysis was additionally conducted for all five prespecified sleep outcomes. Change-score standard deviations were estimated assuming a pre–post correlation of *r* = 0.5 for all trials except Li et al. (58), for which change-score standard deviations were available directly from the published report. For the PSQI global score, this analysis yielded results consistent with the primary post-intervention analysis (MD = 2.46, 95% CI: 1.04 to 3.87, *I*^2^ = 93.2%, *p* < 0.001), with all individual studies demonstrating concordant effect directions and the non-significant effect of Li et al. (58) preserved in both analyses. Results for PSQI-derived component outcomes were similarly consistent in direction with the primary analysis. Full results are presented in Supplementary Appendix 11.

To further evaluate the robustness of the primary finding in the context of small study numbers and considerable heterogeneity, prediction intervals and alternative variance estimators were applied to all five prespecified outcomes. For the PSQI global score, the 95% prediction interval under the DerSimonian–Laird model was −6.98 to 2.12, suggesting that while the average effect favors exercise, the true effect in a new comparable setting may vary considerably. REML estimation yielded a consistent pooled effect (MD = −2.43, 95% CI: −3.68 to −1.18; τ^2^ = 2.50; *I*^2^ = 91.1%), and the Hartung–Knapp adjustment produced a more conservative but still statistically significant confidence interval (MD = −2.43, 95% CI: −3.98 to −0.88; *p* = 0.009). Results for secondary outcomes were consistent in direction with the primary analysis across all three methods. Full results for all outcomes are presented in Supplementary Appendix 12.

Exploratory subgroup analyses were performed according to sex composition and intervention duration. Exercise interventions significantly improved PSQI global score in both all-female trials (MD = −2.50, 95% CI −3.23 to −1.76; *I*^2^ = 0.0%) and mixed-sex trials (MD = −2.45, 95% CI −4.44 to −0.47; *I*^2^ = 95.2%), with no evidence of a between-subgroup difference (Q_between = 0.00, *p* = 0.970).

When stratified by intervention duration, shorter interventions ( ≤ 8 weeks) were associated with a larger effect (MD = −3.29, 95% CI −4.94 to −1.64; *I*^2^ = 84.1%) compared with longer interventions (>8 weeks) (MD = −1.35, 95% CI −2.57 to −0.14; *I*^2^ = 86.0%). The between-subgroup difference approached but did not reach statistical significance (Q_between = 3.43, *p* = 0.064), suggesting a possible attenuation of effects over time.

A *post hoc* exploratory subgroup analysis by exercise modality was also conducted for the PSQI global score (Supplementary Appendix Figure S6.1). Because most exercise-modality subgroups were represented by only one study or comparison, this analysis was intended to explore potential sources of heterogeneity and was not interpreted as evidence of comparative effectiveness between exercise modalities. No exercise-modality subgroup analyses were conducted for the PSQI component outcomes because of the limited number of contributing trials.

Given the limited number of studies within subgroups and the substantial heterogeneity observed, these findings should be interpreted as exploratory and hypothesis-generating.

### Publication bias and certainty of evidence

Visual inspection of the funnel plot for the primary outcome suggested possible asymmetry, and Egger's regression test indicated potential small-study effects for the PSQI global score (*p* = 0.019). However, this finding was interpreted with caution because fewer than 10 comparisons were available, a condition under which formal tests of funnel plot asymmetry have limited statistical power and an elevated risk of false-positive results. For PSQI-derived sleep parameters, publication bias was not formally assessed because each analysis included only three to four trials.

Using the GRADE approach, the certainty of evidence was rated as very low for the PSQI global score, primarily because of serious risk of bias, serious inconsistency, and concerns regarding possible publication bias. Risk of bias was considered serious because six of the seven included trials were judged to have some concerns in RoB 2 domain D4, reflecting the reliance on unblinded participant-reported sleep outcomes. Inconsistency was considered serious because of the considerable between-study heterogeneity (*I*^2^ = 91.7%), while possible publication bias was considered on the basis of funnel plot asymmetry and the result of Egger's regression test, although this judgement was made cautiously given the limited number of comparisons.

The certainty of evidence was rated as very low for sleep duration, sleep latency, and sleep disturbances, and as low for sleep efficiency. The main reasons for downgrading across all secondary outcomes included serious risk of bias attributable to the reliance on unblinded self-reported sleep assessment, substantial inconsistency, and imprecision attributable to the small number of contributing trials. For sleep latency and sleep disturbances, additional downgrading was applied for imprecision given that confidence intervals crossed the line of no effect. The limited number of studies prevented a reliable assessment of publication bias for the secondary outcomes but was not, by itself, treated as an automatic reason for downgrading. Detailed GRADE judgments are presented in Supplementary Appendix 10.

## Discussion

This interpretation is consistent with broader evidence that exercise may improve academic fatigue and sleep quality among university students (27, 64). In addition, observational evidence suggests that sleep quality, stress, and physical activity participation are interrelated in college students, further supporting the relevance of integrated campus-based health-promotion strategies (65).

### Principal findings

In this systematic review and meta-analysis of randomized controlled trials, exercise interventions were associated with a meaningful improvement in PSQI global score among university students compared with non-exercise control conditions, indicating better overall sleep quality. The direction of effect favored exercise across nearly all comparisons, and the primary finding remained robust in leave-one-out sensitivity analyses, suggesting that the overall conclusion was not driven by any single study. For PSQI-derived sleep parameters, improvements appeared most consistent for sleep duration and sleep efficiency, whereas effects on sleep latency and sleep disturbances were directionally favorable but did not reach statistical significance. Exploratory subgroup analyses did not suggest a clear difference by sex composition, while the apparently greater benefit observed in shorter interventions should be interpreted cautiously because of the limited evidence base and substantial heterogeneity. However, the 95% prediction interval for the primary outcome (−6.98 to 2.12) indicates that the true effect of exercise on sleep quality is likely to vary considerably across different settings, populations, and exercise modalities, and a clinically meaningful benefit cannot be assumed in every context. This dispersion of true effects reflects the substantial clinical and methodological heterogeneity among the included trials and underscores the importance of interpreting the pooled estimate as an average rather than a universal effect. Overall, these findings support exercise as a promising, scalable, and low-risk strategy for improving sleep health in university students, although the certainty of evidence remains constrained by inconsistency, imprecision, and possible small-study effects.

### Interpretation in relation to previous evidence

Our findings are broadly consistent with previous systematic reviews and meta-analyses showing that physical activity and structured exercise can improve sleep quality across general adult, older, and clinical populations (33, 34, 60). However, university students should not be regarded simply as a younger subset of the general adult population. This group is exposed to a distinctive constellation of academic, behavioral, and environmental pressures, including irregular schedules, academic fatigue, perceived stress, late-night digital media use, and shared living environments (5, 8, 12, 27).

These factors may help explain the differentiated pattern observed across PSQI components in the present analysis. Exercise produced more consistent benefits for sleep duration and sleep efficiency, parameters that are closely tied to homeostatic sleep pressure and post-sleep-onset consolidation. By contrast, sleep latency and sleep disturbances may be more strongly influenced by pre-sleep cognitive arousal, perceived stress, environmental noise, roommate schedules, and other contextual factors that exercise alone may not fully overcome (1, 4, 12). This pattern is clinically plausible: in university students, sleep problems are often shaped by modifiable behaviors and psychosocial stressors rather than irreversible biological pathology. Exercise may therefore help students maintain longer and more efficient sleep once sleep has begun, while exerting less consistent effects on the behavioral and environmental barriers that delay sleep onset or trigger nocturnal awakenings. This distinction suggests that exercise may be most effective when integrated into broader sleep-health programs rather than delivered as an isolated intervention.

### Potential mechanisms

Several biological and psychological mechanisms may underlie the observed effects. Physiologically, regular exercise may improve sleep by increasing energy expenditure and homeostatic sleep pressure, facilitating post-exercise thermoregulatory changes, supporting circadian regulation, and broadly modulating sleep physiology (22–24, 61). Evidence from systematic reviews and network meta-analyses also suggests that exercise type, dose, intensity, and timing may modify sleep-related benefits, indicating that the effect of exercise on sleep is unlikely to be uniform across all prescriptions (24, 32, 62).

Psychologically, exercise may improve sleep by reducing perceived stress, depressive symptoms, academic fatigue, and ruminative thinking, while enhancing mindfulness, self-efficacy, emotional regulation, and overall mental wellbeing (8, 13, 28, 29, 63). These pathways are particularly relevant to university students, in whom sleep disturbance is frequently maintained by academic pressure, emotional dysregulation, and pre-sleep rumination. Mind–body exercise modalities—such as yoga, Tai Chi, and Pilates—may be especially suitable for campus settings because they combine physical movement with breathing regulation, attentional focus, and relaxation. Nevertheless, the available evidence does not allow firm conclusions about the comparative superiority of one exercise modality over another, and the apparent benefits of different exercise types should therefore be interpreted as hypothesis-generating rather than definitive.

### Implications for campus-based sleep-health promotion

The present findings have practical implications for university health systems. Exercise should not be viewed as a standalone solution for students with severe, persistent, or clinically diagnosed insomnia, but it may serve as an accessible health-promotion strategy and a useful adjunct to behavioral sleep interventions, particularly for students with mild to moderate sleep complaints. This interpretation is consistent with broader evidence that physical activity is associated with better sleep and broader mental-health benefits in student populations (27, 63).

Campus-based programs should prioritize exercise formats that are acceptable, low-cost, supervised when feasible, and compatible with students' academic schedules. Because sleep latency and sleep disturbances were less consistently improved, exercise programs may be more effective when combined with strategies targeting regular sleep timing, evening screen use, stress management, and dormitory sleep environments. The timing of exercise also warrants attention: vigorous activity close to bedtime may adversely affect sleep initiation in some individuals, whereas appropriately timed exercise may support circadian alignment and sleep quality (24). Rather than prescribing exercise generically, universities should therefore consider pragmatic, student-centered programs that integrate exercise prescription with broader sleep-health education.

## Strengths and limitations

This review has several strengths. It focused specifically on university students, a population with a high burden of poor sleep and distinctive behavioral and psychosocial risk factors (5, 27, 63). It included only randomized controlled trials, used the PSQI global score as the primary outcome, examined PSQI-derived sleep parameters separately, and assessed the robustness of the primary finding through leave-one-out sensitivity analyses. This approach allowed a more nuanced interpretation of how exercise may influence different dimensions of sleep quality rather than treating sleep as a single homogeneous construct.

Several limitations should also be acknowledged. First, the evidence base was small, and the included trials varied substantially in exercise modality, intensity, frequency, duration, supervision, baseline sleep status, and outcome reporting. This heterogeneity limits the precision of the pooled estimates and precludes firm conclusions about the optimal exercise prescription. Second, most studies relied on subjective sleep measures, particularly the PSQI. Although the PSQI is widely used in sleep-related research, it is a retrospective self-report instrument and cannot fully capture objective changes in sleep architecture, such as slow-wave sleep, rapid eye movement sleep, or wake after sleep onset (3, 23). Third, blinding participants to exercise allocation is inherently difficult, and expectation effects may have influenced self-reported outcomes. Fourth, although the pooled estimate for the primary outcome was robust across DerSimonian–Laird, REML, and Hartung–Knapp analyses, the wide prediction interval (−6.98 to 2.12) highlights that the average treatment effect may not be reliably reproduced in all future settings, particularly those differing substantially from the included trials in population characteristics, exercise modality, or intervention context. Fifth, possible publication bias and small-study effects cannot be excluded, especially given the limited number of available comparisons. These limitations contributed to low or very low certainty of evidence for several outcomes and should temper the interpretation of the findings.

## Future directions

Future trials should move beyond simple comparisons of exercise vs. no exercise. Large, adequately powered, prospectively registered, and multicenter randomized controlled trials are needed to directly compare different exercise modalities, intensities, frequencies, timings, and durations in university students. Future studies should also report adherence, adverse events, and co-interventions transparently, and should incorporate objective sleep assessments such as actigraphy, wearable-derived sleep metrics, or polysomnography where feasible. Particular attention should be paid to baseline sleep status, psychological distress, academic stress, digital media exposure, exercise timing, and dormitory living conditions, all of which may modify the sleep response to exercise (4, 5, 12, 24).

## Conclusion

In this systematic review and meta-analysis, exercise interventions were associated with improved overall sleep quality, as measured by the PSQI global score, in university students compared with non-exercise control conditions. The benefits appeared most consistent for sleep duration and sleep efficiency, whereas evidence for sleep latency and sleep disturbances remained less certain. Given its accessibility, low cost, and broad physical and mental health benefits, exercise may represent a potentially valuable component of campus-based sleep-health promotion. However, the current evidence is limited by heterogeneity, imprecision, and possible small-study effects. Larger, well-designed randomized trials using standardized and objective sleep outcomes are needed to define the optimal exercise prescription for improving sleep in this population.

## Data Availability

The original contributions presented in the study are included in the article/Supplementary material, further inquiries can be directed to the corresponding author.

## References

[1] Ramar K Malhotra RK Carden KA Martin JL Abbasi-Feinberg F Aurora RN . Sleep is essential to health: an American Academy of Sleep Medicine position statement. J Clin Sleep Med. (2021) 17:2115–9. doi: 10.5664/jcsm.9476 34170250 PMC8494094

[2] Watson NF Badr MS Belenky G Bliwise DL Buxton OM Buysse D . Recommended amount of sleep for a healthy adult: a joint consensus statement of the American Academy of Sleep Medicine and Sleep Research Society. J Clin Sleep Med. (2015) 11:591–2. doi: 10.5664/jcsm.4758 25979105 PMC4442216

[3] Mollayeva T Thurairajah P Burton K Mollayeva S Shapiro CM Colantonio A. The Pittsburgh Sleep Quality Index as a screening tool for sleep dysfunction in clinical and non-clinical samples: a systematic review and meta-analysis. Sleep Med Rev. (2016) 25:52–73. doi: 10.1016/j.smrv.2015.01.009 26163057

[4] Buysse DJ Reynolds CF. 3rd, Monk TH, Berman SR, Kupfer DJ. The Pittsburgh Sleep Quality Index: a new instrument for psychiatric practice and research. Psychiatry Res. (1989) 28:193–213. doi: 10.1016/0165-1781(89)90047-4 2748771

[5] Mbous YPV Nili M Mohamed R Dwibedi N. Psychosocial correlates of insomnia among college students. Prev Chronic Dis. (2022) 19:E60. doi: 10.5888/pcd19.220060 36108290 PMC9480843

[6] Li Y Bai W Zhu B Duan R Yu X Xu W . Prevalence and correlates of poor sleep quality among college students: a cross-sectional survey. Health Qual Life Outcomes. (2020) 18:210. doi: 10.1186/s12955-020-01465-2 32611434 PMC7329462

[7] Ma S Sun Y Jia Y Shi J Sun Y. Impact of poor sleep quality on task switching and reconfiguration process among university students. Behav Sci. (2025) 15:1054. doi: 10.3390/bs15081054 40867411 PMC12383111

[8] Xu L Yan W Hua G Yu Y Wei N Sun L . Effects of physical activity on sleep quality among university students: chain mediation between rumination and depression levels. BMC Psychiatry. (2025) 25:7. doi: 10.1186/s12888-024-06450-3 39748322 PMC11697847

[9] Spyridonidis S Lad D Peters H Ellis J Robinson LJ. Global prevalence of insomnia symptoms in undergraduate university students: a systematic review and meta-analysis. Sleep Adv. (2025) 6:zpaf083. doi: 10.1093/sleepadvances/zpaf083 41377134 PMC12687938

[10] Qanash S Al-Husayni F Falata H Halawa O Jahawi YA Khaled E . Effect of electronic device addiction on sleep quality and academic performance among health care students: cross-sectional study. JMIR Med Educ. (2021) 7:e25662. doi: 10.2196/25662 34612827 PMC8529471

[11] Pham HT Chuang HL Kuo CP Yeh TP Liao WC. Electronic device use before bedtime and sleep quality among university students. Healthcare (Basel). (2021) 9:1091. doi: 10.3390/healthcare9091091 34574865 PMC8466496

[12] Abojedi A Alsheikh Ali AS Basmaji J. Assessing the impact of technology use, social engagement, emotional regulation, and sleep quality among undergraduate students in Jordan: examining the mediating effect of perceived and academic stress. Health Psychol Res. (2023) 11:73348. doi: 10.52965/001c.73348 37025559 PMC10070257

[13] Ye J Jia X Zhang J Guo K. Effect of physical exercise on sleep quality of college students: chain intermediary effect of mindfulness and ruminative thinking. Front Psychol. (2022) 13:987537. doi: 10.3389/fpsyg.2022.987537 36262438 PMC9575948

[14] Edinger JD Arnedt JT Bertisch SM Carney CE Harrington JJ Lichstein KL . Behavioral and psychological treatments for chronic insomnia disorder in adults: an American Academy of Sleep Medicine clinical practice guideline. J Clin Sleep Med. (2021) 17:255–62. doi: 10.5664/jcsm.8986 33164742 PMC7853203

[15] Qaseem A Kansagara D Forciea MA Cooke M Denberg TD. Clinical Guidelines Committee of the American College of Physicians. Management of chronic insomnia disorder in adults: a clinical practice guideline from the American College of Physicians. Ann Intern Med. (2016) 165:125–33. doi: 10.7326/M15-2175 27136449

[16] Sateia MJ Buysse DJ Krystal AD Neubauer DN Heald JL. Clinical practice guideline for the pharmacologic treatment of chronic insomnia in adults: an American Academy of Sleep Medicine clinical practice guideline. J Clin Sleep Med. (2017) 13:307–49. doi: 10.5664/jcsm.6470 27998379 PMC5263087

[17] Rosenberg RP Benca R Doghramji P Roth T. A 2023 update on managing insomnia in primary care: insights from an expert consensus group. Prim Care Companion CNS Disord. (2023) 25:22nr03385. doi: 10.4088/PCC.22nr03385 36705978

[18] Cheung JMY Jarrin DC Ballot O Bharwani AA Morin CM. A systematic review of cognitive behavioral therapy for insomnia implemented in primary care and community settings. Sleep Med Rev. (2019) 44:23–36. doi: 10.1016/j.smrv.2018.11.001 30612061

[19] Koffel E Bramoweth AD Ulmer CS. Increasing access to and utilization of cognitive behavioral therapy for insomnia: a narrative review. J Gen Intern Med. (2018) 33:955–62. doi: 10.1007/s11606-018-4390-1 29619651 PMC5975165

[20] Tadros M Li S Upton E Newby J Werner-Seidler A. Preferences of university students for a psychological intervention designed to improve sleep: focus group study. JMIR Hum Factors. (2023) 10:e44145. doi: 10.2196/44145 37616036 PMC10485721

[21] Tadros M Li S Corkish B Upton E Newby J Werner-Seidler A. Cognitive behavior therapy for insomnia in university students delivered via videoconferencing groups: a pilot study. Behav Sleep Med. (2024) 22:843–56. doi: 10.1080/15402002.2024.2374258 38949071

[22] Kredlow MA Capozzoli MC Hearon BA Calkins AW Otto MW. The effects of physical activity on sleep: a meta-analytic review. J Behav Med. (2015) 38:427–49. doi: 10.1007/s10865-015-9617-6 25596964

[23] Tan X van Egmond LT Cedernaes J Benedict C. The role of exercise-induced peripheral factors in sleep regulation. Mol Metab. (2020) 42:101096. doi: 10.1016/j.molmet.2020.101096 33045432 PMC7585947

[24] Kim N Ka S Park J. Effects of exercise timing and intensity on physiological circadian rhythm and sleep quality: a systematic review. Phys Act Nutr. (2023) 27:52–63. doi: 10.20463/pan.2023.0029 37946447 PMC10636512

[25] De Nys L Anderson K Ofosu EF Ryde GC Connelly J Whittaker AC. The effects of physical activity on cortisol and sleep: a systematic review and meta-analysis. Psychoneuroendocrinology. (2022) 143:105843. doi: 10.1016/j.psyneuen.2022.105843 35777076

[26] Moyers SA Hagger MS. Physical activity and cortisol regulation: a meta-analysis. Biol Psychol. (2023) 179:108548. doi: 10.1016/j.biopsycho.2023.108548 37001634

[27] Fei LL Zhao SX Chen YF. Exercise and sleep health in college students: efficacy, mechanisms, and implications for practice. World J Psychiatry. (2025) 15:108884. doi: 10.5498/wjp.v15.i10.108884 41112608 PMC12531955

[28] He MH Zhao TY Zhu WD Chen J Wu Q Zhang L . The effect of physical exercise on sleep quality in university students: chain mediation of health literacy and life satisfaction. Front Psychol. (2025) 16:1604916. 40667398 10.3389/fpsyg.2025.1604916 PMC12262059

[29] Liu D Tian Y Liu M Yang S. The impact of physical exercise on sleep quality among college students: the chain mediating effects of perceived stress and ruminative thinking. Psychol Res Behav Manag. (2025) 18:361–73. doi: 10.2147/PRBM.S510207 40008246 PMC11853987

[30] Hu YX Liu XM Zhang NX Ma ZY Zhu Z Cao ZB. The effects of resistance are superior to aerobic exercise in improving delayed sleep-wake phase disorder in male college students. Sleep Med. (2025) 128:29–36. doi: 10.1016/j.sleep.2025.01.029 39879677

[31] Wang F Boros S. The effect of physical activity on sleep quality: a systematic review. Eur J Physiother. (2021) 23:11–8. doi: 10.1080/21679169.2019.1623314

[32] Alnawwar MA Alraddadi MI Algethmi RA Almatrafi GA Almehlawi NH Almalki TM. The effect of physical activity on sleep quality and sleep disorder: a systematic review. Cureus. (2023) 15:e43595. doi: 10.7759/cureus.43595 37719583 PMC10503965

[33] Xie Y Liu S Chen XJ Yu HH Yang Y Wang W. Effects of exercise on sleep quality and insomnia in adults: a systematic review and meta-analysis of randomized controlled trials. Front Psychiatry. (2021) 12:664499. doi: 10.3389/fpsyt.2021.664499 34163383 PMC8215288

[34] Memon AR Gupta CC Crowther ME Ferguson SA Tuckwell GA Vincent GE. Sleep and physical activity in university students: a systematic review and meta-analysis. Sleep Med Rev. (2021) 58:101482. doi: 10.1016/j.smrv.2021.101482 33864990

[35] Page MJ McKenzie JE Bossuyt PM Boutron I Hoffmann TC Mulrow CD . The PRISMA 2020 statement: an updated guideline for reporting systematic reviews. BMJ. (2021) 372:n71. doi: 10.1136/bmj.n71 33782057 PMC8005924

[36] Methley AM Campbell S Chew-Graham C McNally R Cheraghi-Sohi S. PICO, PICOS and SPIDER: a comparison study of specificity and sensitivity in three search tools for qualitative systematic reviews. BMC Health Serv Res. (2014) 14:579. doi: 10.1186/s12913-014-0579-0 25413154 PMC4310146

[37] Higgins JPT Thomas J Chandler J Cumpston M Li T Page MJ . Cochrane Handbook for Systematic Reviews of Interventions, Version 6.4 (updated August 2023). London: Cochrane (2023). Available online at: https://training.cochrane.org/handbook (Accessed July 8, 2026).

[38] de Vries JD van Hooff ML Geurts SA Kompier MA. Exercise as an intervention to reduce study-related fatigue among university students: a two-arm parallel randomized controlled trial. PLoS ONE. (2016) 11:e0152137. doi: 10.1371/journal.pone.0152137 27031610 PMC4816334

[39] Zhou X Li F Feng N Teng Y Yu Q. Effects of brisk walking and Tai Chi interventions on sleep quality in university students with insomnia. Front Public Health. (2026) 13:1732579. doi: 10.3389/fpubh.2025.1732579 41607891 PMC12834728

[40] Sterne JAC Savović J Page MJ Elbers RG Blencowe NS Boutron I . RoB 2: a revised tool for assessing risk of bias in randomised trials. BMJ. (2019) 366:l4898. doi: 10.1136/bmj.l4898 31462531

[41] McGuinness LA Higgins JPT. Risk-of-bias VISualization (robvis): an R package and Shiny web app for visualizing risk-of-bias assessments. Res Synth Methods. (2021) 12:55–61. doi: 10.1002/jrsm.1411 32336025

[42] Guyatt GH Oxman AD Vist GE Kunz R Falck-Ytter Y Alonso-Coello P . GRADE: an emerging consensus on rating quality of evidence and strength of recommendations. BMJ. (2008) 336:924–6. doi: 10.1136/bmj.39489.470347.AD 18436948 PMC2335261

[43] Guyatt GH Oxman AD Vist G Kunz R Brozek J Alonso-Coello P . GRADE guidelines: 4. Rating the quality of evidence—study limitations (risk of bias). J Clin Epidemiol. (2011) 64:407–15. doi: 10.1016/j.jclinepi.2010.07.017 21247734

[44] Guyatt GH Oxman AD Kunz R Woodcock J Brozek J Helfand M . GRADE guidelines: 7. Rating the quality of evidence—inconsistency. J Clin Epidemiol. (2011) 64:1294–302. doi: 10.1016/j.jclinepi.2011.03.017 21803546

[45] Guyatt GH Oxman AD Kunz R Woodcock J Brozek J Helfand M . GRADE guidelines: 8. Rating the quality of evidence—indirectness. J Clin Epidemiol. (2011) 64:1303–10. doi: 10.1016/j.jclinepi.2011.04.014 21802903

[46] Guyatt GH Oxman AD Kunz R Brozek J Alonso-Coello P Rind D . GRADE guidelines: 6. Rating the quality of evidence—imprecision. J Clin Epidemiol. (2011) 64:1283–93. doi: 10.1016/j.jclinepi.2011.01.012 21839614

[47] Guyatt GH Oxman AD Montori V Vist G Kunz R Brozek J . GRADE guidelines: 5. Rating the quality of evidence—publication bias. J Clin Epidemiol. (2011) 64:1277–82. doi: 10.1016/j.jclinepi.2011.01.011 21802904

[48] GRADEpro GDT: GRADEpro Guideline Development Tool. Hamilton, ON: McMaster University and Evidence Prime (2024). Available online at: https://www.gradepro.org (Accessed July 8, 2026).

[49] Harris RJ Bradburn MJ Deeks JJ Harbord RM Altman DG Sterne JAC. metan: fixed- and random-effects meta-analysis. Stata J. (2008) 8:3–28. doi: 10.1177/1536867X0800800102

[50] DerSimonian R Laird N. Meta-analysis in clinical trials. Control Clin Trials. (1986) 7:177–88. doi: 10.1016/0197-2456(86)90046-2 3802833

[51] Higgins JPT Thompson SG Deeks JJ Altman DG. Measuring inconsistency in meta-analyses. BMJ. (2003) 327:557–60. doi: 10.1136/bmj.327.7414.557 12958120 PMC192859

[52] Patsopoulos NA Evangelou E Ioannidis JPA. Sensitivity of between-study heterogeneity in meta-analysis: proposed metrics and empirical evaluation. Int J Epidemiol. (2008) 37:1148–57. doi: 10.1093/ije/dyn065 18424475 PMC6281381

[53] Egger M Davey Smith G Schneider M Minder C. Bias in meta-analysis detected by a simple, graphical test. BMJ. (1997) 315:629–34. doi: 10.1136/bmj.315.7109.629 9310563 PMC2127453

[54] Sterne JAC Sutton AJ Ioannidis JPA Terrin N Jones DR Lau J . Recommendations for examining and interpreting funnel plot asymmetry in meta-analyses of randomised controlled trials. BMJ. (2011) 343:d4002. doi: 10.1136/bmj.d4002 21784880

[55] Fu J Zhang W Xu X Yan H Xu Z Su X . The impact of 4-week high-intensity interval training on mental health and sleep quality in female college students with normal weight obesity: a randomized controlled trial. J Transl Med. (2025) 23:1234. doi: 10.1186/s12967-025-07276-7 41199311 PMC12590694

[56] Ezati M Keshavarz M Barandouzi ZA Montazeri A. The effect of regular aerobic exercise on sleep quality and fatigue among female student dormitory residents. BMC Sports Sci Med Rehabil. (2020) 12:44. doi: 10.1186/s13102-020-00190-z 32774864 PMC7405354

[57] Amzajerdi A Keshavarz M Ezati M Sarvi F. The effect of Pilates exercises on sleep quality and fatigue among female students dormitory residents. BMC Sports Sci Med Rehabil. (2023) 15:67. doi: 10.1186/s13102-023-00675-7 37101195 PMC10134533

[58] Li M Fang Q Li J Zheng X Tao J Yan X . The effect of Chinese traditional exercise—Baduanjin on physical and psychological well-being of college students: a randomized controlled trial. PLoS ONE. (2015) 10:e0130544. doi: 10.1371/journal.pone.0130544 26158769 PMC4497728

[59] Iyer S Bhat R Gulati K Bhargav H. Dawn vs. dusk: yoga practice timing shapes sleep, mood and well-being in young adults—results of a randomized controlled trial. J Ayurveda Integr Med. (2026) 17:101314. doi: 10.1016/j.jaim.2025.101314 41831285 PMC13000548

[60] Uchida S Shioda K Morita Y Kubota C Ganeko M Takeda N. Exercise effects on sleep physiology. Front Neurol. (2012) 3:48. doi: 10.3389/fneur.2012.00048 22485106 PMC3317043

[61] Wang H Xin X Pan Y. The best approaches and doses of exercise for improving sleep quality: a network meta-analysis and dose-response relationship study. BMC Public Health. (2025) 25:1371. doi: 10.1186/s12889-025-22570-1 40217183 PMC11987399

[62] Li W Chen J Li M Smith AP Fan J. The effect of exercise on academic fatigue and sleep quality among university students. Front Psychol. (2022) 13:1025280. doi: 10.3389/fpsyg.2022.1025280 36337542 PMC9634171

[63] Wang H Zhao S Fang T Xiao L Yin D Sun Z. Sleep quality and stress as influences on college students' physical activity participation: a cross-sectional study. Front Public Health. (2025) 13:1640974. doi: 10.3389/fpubh.2025.1640974 41158584 PMC12554725

[64] Banno M Harada Y Taniguchi M Tobita R Tsujimoto H Tsujimoto Y . Exercise can improve sleep quality: a systematic review and meta-analysis. PeerJ. (2018) 6:e5172. doi: 10.7717/peerj.5172 30018855 PMC6045928

[65] Bu ZJ Liu FS Shahjalal M Song YK Li MC Zhuo RE . Effects of various exercise interventions in insomnia patients: a systematic review and network meta-analysis. BMJ Evid Based Med. (2026) 31:102–13. doi: 10.1136/bmjebm-2024-113512 40664502

