# Supplementary material for: Exercise interventions to improve sleep quality in university students: a systematic review and meta-analysis of randomized controlled trials
Source: Front Public Health. 2026 Jul 21;14:1871771. doi: 10.3389/fpubh.2026.1871771 (PMC13435322; doi:10.3389/fpubh.2026.1871771)
Supplement: Supplementary file 1 [file Data_Sheet_1.docx]

**Supplementary Appendix**

**Exercise interventions for improving sleep quality in university students: implications for public health from a meta-analysis of randomized controlled trials**

**Table of contents**

**[Appendix 1:](#_Toc1346393294)** [Checklist for Reporting Systematic Reviews (PRISMA 2020)	2](#_Toc1346393294)

**[Appendix 2:](#_Toc796903819)** [Search strategy](#_Toc796903819)	8

**[Appendix 3:](#_Toc1854463241)** [Risk of bias assessment	1](#_Toc1854463241)8

**[Appendix 4:](#_Toc1533522576)** [Detailed forest plot	1](#_Toc1533522576)9

**[Appendix 5:](#_Toc1962687185)** [Leave-one-out sensitivity analysis	21](#_Toc1962687185)

**[Appendix 6:](#_Toc1534700375)** [Subgroup analysis by exercise type](#_Toc1534700375)

[24](#_Toc1534700375)

**[Appendix 7:](#_Toc283118508)** [Exploratory funnel plots	2](#_Toc283118508)5

**[Appendix 8:](#_Toc1696485851)** [Subgroup analyses for PSQI global score](#_Toc1696485851)

[.	2](#_Toc1696485851)7

**[Appendix 9:](#_Toc697316538)** [Exploratory Egger's test for publication bias across sleep quality outcomes](#_Toc697316538)

[.	2](#_Toc697316538)8

**[Appendix 10:](#_Toc980792487)** [GRADE assessment](#_Toc980792487)

[.	29](#_Toc980792487)

**[Appendix 11:](#_Toc1533522576)** [Sensitivity analysis using change-from-baseline scores](#_Toc1533522576)

[30](#_Toc1533522576)

**[Appendix 12:](#_Toc980792487)** [Robustness checks for the primary outcome — prediction interval, REML estimation, and Hartung–Knapp correction	4](#_Toc980792487)2

**Appendix 1: Checklist for Reporting Systematic Reviews (PRISMA 2020)**

| **Section/Topic** | **Item #** | **Checklist Item** | **Reported on Page #** |
| --- | --- | --- | --- |
| **TITLE** |  |  |  |
| Title | 1 | Identify the report as a systematic review. | ***1*** |
|  |  |  |  |
| **ABSTRACT** |  |  | ***1*** |
| Structured summary | 2 | See the PRISMA 2020 for Abstracts checklist (Background, Methods, Results, Discussion, Other). |  |
|  |  |  |  |
| **INTRODUCTION** |  |  |  |
| Rationale | 3 | Describe the rationale for the review in the context of existing knowledge. | ***2-4*** |
| Objectives | 4 | Provide an explicit statement of the objective(s) or question(s) the review addresses, with reference to PICOS (Population, Intervention, Comparator, Outcomes, Study design). | **4** |
|  |  |  |  |
| **METHODS** |  |  |  |
| Eligibility criteria | 5 | Specify the inclusion and exclusion criteria for the review and how studies were grouped for the syntheses. | **5-6** |
| Information sources | 6 | Specify all databases, registers, websites, organizations, reference lists, and other sources searched or consulted to identify studies. Specify the date when each source was last searched or consulted. | ***5*** |
| Search strategy | 7 | Present the full search strategies for all databases, registers, and websites, including any filters and limits used. | **5; Supplementary Tables S1–S7** |
| Selection process | 8 | Specify the methods used to decide whether a study met the inclusion criteria, including how many reviewers screened each record and each report retrieved, whether they worked independently, and any automation tools used in the process. | **6** |
| Data collection process | 9 | Specify the methods used to collect data from reports, including how many reviewers collected data, whether they worked independently, any automation tools used, and any processes for obtaining or confirming data from study investigators. | **6-7** |
| Data items | 10a | List and define all outcomes for which data were sought. Specify whether all results that were compatible with each outcome domain in each study were sought, and if not, how the results were chosen. | **6-7** |
|  | 10b | List and define all other variables for which data were sought (e.g., participant characteristics, intervention characteristics, funding sources). Describe any assumptions made about missing or unclear information. | **6-7** |
| **Study risk of bias assessment** | **11** | Specify the methods used to assess risk of bias in the included studies, including details of the tool(s) used (RoB 2), how many reviewers assessed each study, whether they worked independently, and any automation tools used. | ***7*** |
| Effect measures | 12 | Specify for each outcome the effect measure(s) (e.g., mean difference) used in the synthesis or presentation of results. | **9-10** |
| Synthesis methods | 13a | Describe the processes used to decide which studies were eligible for each synthesis (e.g., tabulating study intervention characteristics and comparing against the planned groups for each synthesis). | **9-10** |
|  | 13b | Describe any methods required to prepare the data for presentation or synthesis, such as handling of missing summary statistics or data conversions. | **9-10** |
|  | 13c | Describe any methods used to tabulate or visually display results of individual studies and syntheses. | **9-10** |
|  | 13d | Describe any methods used to synthesize results and provide a rationale for the choice(s). If meta-analysis was performed, describe the model(s), method(s) to identify the presence and extent of statistical heterogeneity, and software package(s) used. | **9-10** |
|  | 13e | Describe any methods used to explore possible causes of heterogeneity among study results (e.g., subgroup analysis, meta-regression). | **10** |
|  | 13f | Describe any sensitivity analyses conducted to assess the robustness of the synthesized results. | **10** |
| Reporting bias assessment | 14 | Describe any methods used to assess risk of bias due to missing results in a synthesis (arising from reporting biases). | **10** |
| Certainty assessment | 15 | Describe any methods used to assess certainty (or confidence) in the body of evidence for an outcome (GRADE). | **8** |
| **RESULTS†** |  |  |  |
| Study selection | 16a | Describe the results of the search and selection process, from the number of records identified in the search to the number of studies included in the review, ideally using a flow diagram. | **11** |
|  | 16b | Cite studies that might appear to meet the inclusion criteria, but which were excluded, and explain why they were excluded. | ***11*** |
| Study characteristics | 17 | Cite each included study and present its characteristics. | ***11-12*** |
| Risk of bias in studies | 18 | Present assessments of risk of bias for each included study. | **12-13** |
| Results of individual studies | 19 | For all outcomes, present, for each study: (a) summary statistics for each group (where appropriate), and (b) an effect estimate and its precision (e.g., 95% confidence interval), ideally using structured tables or plots. | **13-16** |
| Results of syntheses | 20a | For each synthesis, briefly summarize the characteristics and risk of bias among contributing studies. | **13-16** |
|  | 20b | Present results of all statistical syntheses conducted. If meta-analysis was performed, present for each the summary estimate and its precision (e.g., 95% confidence interval) and measures of statistical heterogeneity. If comparing groups, describe the direction of the effect. | **13-16** |
|  | 20c | Present results of all investigations of possible causes of heterogeneity among study results. | **16-17** |
|  | 20d | Present results of all sensitivity analyses conducted to assess the robustness of the synthesized results. | **16-17** |
| Reporting biases | 21 | Present assessments of risk of bias due to missing results (arising from reporting biases) for each synthesis assessed. | ***17*** |
| Certainty of evidence | 22 | Present assessments of certainty (or confidence) in the body of evidence for each outcome assessed. | **17; Supplementary Appendix 10** |
| **DISCUSSION** |  |  |  |
| Discussion | 23a | Provide a general interpretation of the results in the context of other evidence. | **18–19** |
|  | 23b | Discuss any limitations of the evidence included in the review. | **20–21** |
|  | 23c | Discuss any limitations of the review processes used. | **20–21** |
|  | 23d | Discuss implications of the results for practice, policy, and future research. | **19–21** |
| **OTHER INFORMATION** |  |  |  |
| Registration and protocol | 24a | Describe sources of funding and other support for the review, the role of the funders, and any potential conflicts of interest relevant to the interventions included in the network. | ***22*** |
|  | 24b | Indicate where the review protocol can be accessed, or state that a protocol was not prepared. | ***22*** |
|  | 24c | Describe and explain any amendments to information provided at registration or in the protocol. | **N/A** |
| Support | 25 | Describe sources of financial or non-financial support for the review, and the role of the funders or sponsors in the review. | **22** |
| Competing interests | 26 | Declare any competing interests of review authors. | **22** |
| Availability of data, code and other materials | 27 | Report which of the following are publicly available and where they can be found: template data collection forms; data extracted from included studies; data used for all analyses; analytic code; any other materials used in the review. | **N/A** |

**Appendix 2: Search strategy**

**Table S1.** Search strategy of PubMed

| **#** | **Searches** |
| --- | --- |
| 1 | ("Students"[Mesh]) OR ("Universities"[Mesh]) |
| 2 | ((Universit*[Title/Abstract]) OR (college student*[Title/Abstract]) OR (undergraduate*[Title/Abstract]) OR (postgraduate*[Title/Abstract]) OR (student*[Title/Abstract])) |
| 3 | (("Students"[Mesh]) OR ("Universities"[Mesh])) OR (((((Universit*[Title/Abstract]) OR (college student*[Title/Abstract])) OR (undergraduate*[Title/Abstract])) OR (postgraduate*[Title/Abstract])) OR (student*[Title/Abstract])) |
| 4 | ("Exercise"[Mesh]) OR ("Sports"[Mesh]) OR ("Motor Activity"[Mesh]) |
| 5 | ((((((((((Exercise*[Title/Abstract]) OR (physical activity[Title/Abstract])) OR (physical exercise*[Title/Abstract])) OR (aerobic exercise*[Title/Abstract])) OR (resistance training*[Title/Abstract])) OR (exercise training*[Title/Abstract])) OR (sport*[Title/Abstract])) OR (yoga[Title/Abstract])) OR (tai chi[Title/Abstract])) OR (qigong[Title/Abstract])) OR (training[Title/Abstract]) |
| 6 | (("Exercise"[Mesh]) OR ("Sports"[Mesh]) OR ("Motor Activity"[Mesh])) OR (((((((((((Exercise*[Title/Abstract]) OR (physical activity[Title/Abstract])) OR (physical exercise*[Title/Abstract])) OR (aerobic exercise*[Title/Abstract])) OR (resistance training*[Title/Abstract])) OR (exercise training*[Title/Abstract])) OR (sport*[Title/Abstract])) OR (yoga[Title/Abstract])) OR (tai chi[Title/Abstract])) OR (qigong[Title/Abstract])) OR (training[Title/Abstract])) |
| 7 | ("Sleep"[Mesh]) OR ("Sleep Initiation and Maintenance Disorders"[Mesh]) OR ("Sleep Wake Disorders"[Mesh]) |
| 8 | (((((sleep quality[Title/Abstract]) OR (sleep[Title/Abstract])) OR (insomnia[Title/Abstract])) OR (Pittsburgh Sleep Quality Index[Title/Abstract])) OR (PSQI[Title/Abstract])) |
| 9 | (("Sleep"[Mesh]) OR ("Sleep Initiation and Maintenance Disorders"[Mesh]) OR ("Sleep Wake Disorders"[Mesh])) OR ((((((sleep quality[Title/Abstract]) OR (sleep[Title/Abstract])) OR (insomnia[Title/Abstract])) OR (Pittsburgh Sleep Quality Index[Title/Abstract])) OR (PSQI[Title/Abstract]))) |
| 10 | #3 AND #6 AND #9 |
| 11 | (((("meta-analysis"[Publication Type]) OR ("systematic review"[Publication Type])) OR ("meta-analysis"[Title/Abstract])) OR ("systematic review"[Title/Abstract])) |
| 12 | #10 NOT #11 |
| 13 | ((((((((randomized controlled trial[Publication Type]) OR (controlled clinical trial[Publication Type])) OR (clinical trials as topic[MeSH Terms])) OR (random allocation[MeSH Terms])) OR (randomized controlled trials as topic[MeSH Terms])) OR (randomly[Title/Abstract])) OR (randomized[Title/Abstract])) OR (trial[Title/Abstract])) OR (placebo[Title/Abstract]) |
| 14 | #12 AND #13 |

**Table S2.** Search strategy of Web of Science

| **#** | **Searches** |
| --- | --- |
| 1 | (TI=(Universit* OR "college student*" OR undergraduate* OR postgraduate* OR student*) OR AB=(Universit* OR "college student*" OR undergraduate* OR postgraduate* OR student*)) |
| 2 | (TI=(Exercise* OR "physical activity" OR "physical exercise*" OR "aerobic exercise*" OR "resistance training*" OR "exercise training*" OR sport* OR yoga OR "tai chi" OR qigong OR training) OR AB=(Exercise* OR "physical activity" OR "physical exercise*" OR "aerobic exercise*" OR "resistance training*" OR "exercise training*" OR sport* OR yoga OR "tai chi" OR qigong OR training)) |
| 3 | (TI=("sleep quality" OR sleep OR insomnia OR "Pittsburgh Sleep Quality Index" OR PSQI) OR AB=("sleep quality" OR sleep OR insomnia OR "Pittsburgh Sleep Quality Index" OR PSQI)) |
| 4 | #1 AND #2 AND #3 |
| 5 | (TI=("meta-analysis" OR "systematic review") OR AB=("meta-analysis" OR "systematic review")) |
| 6 | #4 NOT #5 |
| 7 | (TI=("randomized controlled trial*" OR "controlled clinical trial*" OR "randomized clinical trial*" OR randomly OR randomized OR trial OR placebo OR RCT) OR AB=("randomized controlled trial*" OR "controlled clinical trial*" OR "randomized clinical trial*" OR randomly OR randomized OR trial OR placebo OR RCT)) |
| 8 | #6 AND #7 |

**Table S3.** Search strategy of Embase

| **#** | **Searches** |
| --- | --- |
| 1 | 'student'/exp OR 'university'/exp OR 'college'/exp |
| 2 | (Universit*:ti,ab OR 'college student*':ti,ab OR undergraduate*:ti,ab OR postgraduate*:ti,ab OR student*:ti,ab) |
| 3 | ('student'/exp OR 'university'/exp OR 'college'/exp) OR (Universit*:ti,ab OR 'college student*':ti,ab OR undergraduate*:ti,ab OR postgraduate*:ti,ab OR student*:ti,ab) |
| 4 | 'exercise'/exp OR 'sport'/exp OR 'physical activity'/exp |
| 5 | (exercise*:ti,ab OR 'physical activity':ti,ab OR 'physical exercise*':ti,ab OR 'aerobic exercise*':ti,ab OR 'resistance training*':ti,ab OR 'exercise training*':ti,ab OR sport*:ti,ab OR yoga:ti,ab OR 'tai chi':ti,ab OR qigong:ti,ab OR training:ti,ab) |
| 6 | ('exercise'/exp OR 'sport'/exp OR 'physical activity'/exp) OR (exercise*:ti,ab OR 'physical activity':ti,ab OR 'physical exercise*':ti,ab OR 'aerobic exercise*':ti,ab OR 'resistance training*':ti,ab OR 'exercise training*':ti,ab OR sport*:ti,ab OR yoga:ti,ab OR 'tai chi':ti,ab OR qigong:ti,ab OR training:ti,ab) |
| 7 | 'sleep'/exp OR 'insomnia'/exp OR 'sleep disorder'/exp |
| 8 | ('sleep quality':ti,ab OR sleep:ti,ab OR insomnia:ti,ab OR 'Pittsburgh Sleep Quality Index':ti,ab OR PSQI:ti,ab) |
| 9 | ('sleep'/exp OR 'insomnia'/exp OR 'sleep disorder'/exp) OR ('sleep quality':ti,ab OR sleep:ti,ab OR insomnia:ti,ab OR 'Pittsburgh Sleep Quality Index':ti,ab OR PSQI:ti,ab) |
| 10 | #3 AND #6 AND #9 |
| 11 | ('meta-analysis'/exp OR 'systematic review'/exp) OR ('meta-analysis':ti,ab OR 'systematic review':ti,ab) |
| 12 | #10 NOT #11 |
| 13 | ('randomized controlled trial'/exp OR 'controlled clinical trial'/exp) OR ('randomized controlled trial*':ti,ab OR 'controlled clinical trial*':ti,ab OR 'randomized clinical trial*':ti,ab OR randomly:ti,ab OR randomized:ti,ab OR trial:ti,ab OR placebo:ti,ab OR RCT:ti,ab) |
| 14 | #12 AND #13 |

**Table S4.** Search strategy of Cochrane Central Register of Controlled Trials

| **#** | **Searches** |
| --- | --- |
| 1 | (MeSH descriptor: [Students] explode all trees) OR (MeSH descriptor: [Universities] explode all trees) |
| 2 | ((Universit*:ti,ab) OR ("college student*":ti,ab) OR (undergraduate*:ti,ab) OR (postgraduate*:ti,ab) OR (student*:ti,ab)) |
| 3 | ((MeSH descriptor: [Students] explode all trees) OR (MeSH descriptor: [Universities] explode all trees)) OR (((((Universit*:ti,ab) OR ("college student*":ti,ab)) OR (undergraduate*:ti,ab)) OR (postgraduate*:ti,ab)) OR (student*:ti,ab)) |
| 4 | (MeSH descriptor: [Exercise] explode all trees) OR (MeSH descriptor: [Sports] explode all trees) OR (MeSH descriptor: [Motor Activity] explode all trees) |
| 5 | (((((((((((Exercise*:ti,ab) OR ("physical activity":ti,ab)) OR ("physical exercise*":ti,ab)) OR ("aerobic exercise*":ti,ab)) OR ("resistance training*":ti,ab)) OR ("exercise training*":ti,ab)) OR (sport*:ti,ab)) OR (yoga:ti,ab)) OR ("tai chi":ti,ab)) OR (qigong:ti,ab)) OR (training:ti,ab)) |
| 6 | ((MeSH descriptor: [Exercise] explode all trees) OR (MeSH descriptor: [Sports] explode all trees) OR (MeSH descriptor: [Motor Activity] explode all trees)) OR (((((((((((Exercise*:ti,ab) OR ("physical activity":ti,ab)) OR ("physical exercise*":ti,ab)) OR ("aerobic exercise*":ti,ab)) OR ("resistance training*":ti,ab)) OR ("exercise training*":ti,ab)) OR (sport*:ti,ab)) OR (yoga:ti,ab)) OR ("tai chi":ti,ab)) OR (qigong:ti,ab)) OR (training:ti,ab)) |
| 7 | (MeSH descriptor: [Sleep] explode all trees) OR (MeSH descriptor: [Sleep Initiation and Maintenance Disorders] explode all trees) OR (MeSH descriptor: [Sleep Wake Disorders] explode all trees) |
| 8 | (((((("sleep quality":ti,ab) OR (sleep:ti,ab)) OR (insomnia:ti,ab)) OR ("Pittsburgh Sleep Quality Index":ti,ab)) OR (PSQI:ti,ab))) |
| 9 | (MeSH descriptor: [Sleep] explode all trees) OR (MeSH descriptor: [Sleep Initiation and Maintenance Disorders] explode all trees) OR (MeSH descriptor: [Sleep Wake Disorders] explode all trees)) OR (((((("sleep quality":ti,ab) OR (sleep:ti,ab)) OR (insomnia:ti,ab)) OR ("Pittsburgh Sleep Quality Index":ti,ab)) OR (PSQI:ti,ab))) |
| 10 | #3 AND #6 AND #9 |
| 11 | (((((("randomized controlled trial*":ti,ab) OR ("controlled clinical trial*":ti,ab)) OR ("randomized clinical trial*":ti,ab)) OR (randomly:ti,ab)) OR (randomized:ti,ab)) OR (trial:ti,ab) OR (placebo:ti,ab) OR (RCT:ti,ab) |
| 12 | #10 AND #11 |

**Table S5.** Search strategy of CINAHL

| **#** | **Searches** |
| --- | --- |
| 1 | (MH "Students+") OR (MH "Universities+") OR (MH "Colleges+") |
| 2 | ((TI(Universit*) OR AB(Universit*)) OR (TI("college student*") OR AB("college student*")) OR (TI(undergraduate*) OR AB(undergraduate*)) OR (TI(postgraduate*) OR AB(postgraduate*)) OR (TI(student*) OR AB(student*))) |
| 3 | ((MH "Students+") OR (MH "Universities+") OR (MH "Colleges+")) OR (((((TI(Universit*) OR AB(Universit*)) OR (TI("college student*") OR AB("college student*"))) OR (TI(undergraduate*) OR AB(undergraduate*))) OR (TI(postgraduate*) OR AB(postgraduate*))) OR (TI(student*) OR AB(student*))) |
| 4 | (MH "Exercise+") OR (MH "Sports+") OR (MH "Physical Activity+") |
| 5 | ((((((((((TI(Exercise*) OR AB(Exercise*)) OR (TI("physical activity") OR AB("physical activity"))) OR (TI("physical exercise*") OR AB("physical exercise*"))) OR (TI("aerobic exercise*") OR AB("aerobic exercise*"))) OR (TI("resistance training*") OR AB("resistance training*"))) OR (TI("exercise training*") OR AB("exercise training*"))) OR (TI(sport*) OR AB(sport*))) OR (TI(yoga) OR AB(yoga))) OR (TI("tai chi") OR AB("tai chi"))) OR (TI(qigong) OR AB(qigong))) OR (TI(training) OR AB(training)) |
| 6 | ((MH "Exercise+") OR (MH "Sports+") OR (MH "Physical Activity+")) OR (((((((((((TI(Exercise*) OR AB(Exercise*)) OR (TI("physical activity") OR AB("physical activity"))) OR (TI("physical exercise*") OR AB("physical exercise*"))) OR (TI("aerobic exercise*") OR AB("aerobic exercise*"))) OR (TI("resistance training*") OR AB("resistance training*"))) OR (TI("exercise training*") OR AB("exercise training*"))) OR (TI(sport*) OR AB(sport*))) OR (TI(yoga) OR AB(yoga))) OR (TI("tai chi") OR AB("tai chi"))) OR (TI(qigong) OR AB(qigong))) OR (TI(training) OR AB(training)) |
| 7 | (MH "Sleep+") OR (MH "Sleep Disorders+") OR (MH "Insomnia+") |
| 8 | (((((TI("sleep quality") OR AB("sleep quality")) OR (TI(sleep) OR AB(sleep))) OR (TI(insomnia) OR AB(insomnia))) OR (TI("Pittsburgh Sleep Quality Index") OR AB("Pittsburgh Sleep Quality Index"))) OR (TI(PSQI) OR AB(PSQI))) |
| 9 | ((MH "Sleep+") OR (MH "Sleep Disorders+") OR (MH "Insomnia+")) OR ((((((TI("sleep quality") OR AB("sleep quality")) OR (TI(sleep) OR AB(sleep))) OR (TI(insomnia) OR AB(insomnia))) OR (TI("Pittsburgh Sleep Quality Index") OR AB("Pittsburgh Sleep Quality Index"))) OR (TI(PSQI) OR AB(PSQI)))) |
| 10 | #3 AND #6 AND #9 |
| 11 | (MH "Randomized Controlled Trials+") OR (MH "Clinical Trials+") OR (MH "Random Assignment+") |
| 12 | ((((((TI("randomized controlled trial*") OR AB("randomized controlled trial*")) OR (TI("controlled clinical trial*") OR AB("controlled clinical trial*"))) OR (TI("randomized clinical trial*") OR AB("randomized clinical trial*"))) OR (TI(randomly) OR AB(randomly))) OR (TI(randomized) OR AB(randomized))) OR (TI(trial) OR AB(trial))) OR (TI(placebo) OR AB(placebo)) OR (TI(RCT) OR AB(RCT)) |
| 13 | ((MH "Randomized Controlled Trials+") OR (MH "Clinical Trials+") OR (MH "Random Assignment+")) OR ((((((TI("randomized controlled trial*") OR AB("randomized controlled trial*")) OR (TI("controlled clinical trial*") OR AB("controlled clinical trial*"))) OR (TI("randomized clinical trial*") OR AB("randomized clinical trial*"))) OR (TI(randomly) OR AB(randomly))) OR (TI(randomized) OR AB(randomized))) OR (TI(trial) OR AB(trial))) OR (TI(placebo) OR AB(placebo)) OR (TI(RCT) OR AB(RCT)) |
| 14 | #10 AND #13 |

**Table S6.** Search strategy of PsycINFO

| **#** | **Searches** |
| --- | --- |
| 1 | (DE "College Students") OR (DE "University Students") OR (DE "Graduate Students") OR (DE "Undergraduate Students") |
| 2 | ((TI(Universit*) OR AB(Universit*)) OR (TI("college student*") OR AB("college student*")) OR (TI(undergraduate*) OR AB(undergraduate*)) OR (TI(postgraduate*) OR AB(postgraduate*)) OR (TI(student*) OR AB(student*))) |
| 3 | ((DE "College Students") OR (DE "University Students") OR (DE "Graduate Students") OR (DE "Undergraduate Students")) OR (((((TI(Universit*) OR AB(Universit*)) OR (TI("college student*") OR AB("college student*"))) OR (TI(undergraduate*) OR AB(undergraduate*))) OR (TI(postgraduate*) OR AB(postgraduate*))) OR (TI(student*) OR AB(student*))) |
| 4 | (DE "Exercise") OR (DE "Physical Activity") OR (DE "Athletic Training") OR (DE "Aerobic Exercise") |
| 5 | ((((((((((TI(Exercise*) OR AB(Exercise*)) OR (TI("physical activity") OR AB("physical activity"))) OR (TI("physical exercise*") OR AB("physical exercise*"))) OR (TI("aerobic exercise*") OR AB("aerobic exercise*"))) OR (TI("resistance training*") OR AB("resistance training*"))) OR (TI("exercise training*") OR AB("exercise training*"))) OR (TI(sport*) OR AB(sport*))) OR (TI(yoga) OR AB(yoga))) OR (TI("tai chi") OR AB("tai chi"))) OR (TI(qigong) OR AB(qigong))) OR (TI(training) OR AB(training)) |
| 6 | ((DE "Exercise") OR (DE "Physical Activity") OR (DE "Athletic Training") OR (DE "Aerobic Exercise")) OR (((((((((((TI(Exercise*) OR AB(Exercise*)) OR (TI("physical activity") OR AB("physical activity"))) OR (TI("physical exercise*") OR AB("physical exercise*"))) OR (TI("aerobic exercise*") OR AB("aerobic exercise*"))) OR (TI("resistance training*") OR AB("resistance training*"))) OR (TI("exercise training*") OR AB("exercise training*"))) OR (TI(sport*) OR AB(sport*))) OR (TI(yoga) OR AB(yoga))) OR (TI("tai chi") OR AB("tai chi"))) OR (TI(qigong) OR AB(qigong))) OR (TI(training) OR AB(training)) |
| 7 | (DE "Sleep") OR (DE "Sleep Disorders") OR (DE "Insomnia") OR (DE "Sleep Quality") |
| 8 | (((((TI("sleep quality") OR AB("sleep quality")) OR (TI(sleep) OR AB(sleep))) OR (TI(insomnia) OR AB(insomnia))) OR (TI("Pittsburgh Sleep Quality Index") OR AB("Pittsburgh Sleep Quality Index"))) OR (TI(PSQI) OR AB(PSQI))) |
| 9 | ((DE "Sleep") OR (DE "Sleep Disorders") OR (DE "Insomnia") OR (DE "Sleep Quality")) OR ((((((TI("sleep quality") OR AB("sleep quality")) OR (TI(sleep) OR AB(sleep))) OR (TI(insomnia) OR AB(insomnia))) OR (TI("Pittsburgh Sleep Quality Index") OR AB("Pittsburgh Sleep Quality Index"))) OR (TI(PSQI) OR AB(PSQI)))) |
| 10 | #3 AND #6 AND #9 |
| 11 | (DE "Systematic Review") OR (DE "Meta Analysis") |
| 12 | ((TI("meta-analysis") OR AB("meta-analysis")) OR (TI("systematic review") OR AB("systematic review"))) |
| 13 | ((DE "Systematic Review") OR (DE "Meta Analysis")) OR ((TI("meta-analysis") OR AB("meta-analysis")) OR (TI("systematic review") OR AB("systematic review"))) |
| 14 | #10 NOT #13 |
| 15 | (DE "Randomized Controlled Trials") OR (DE "Clinical Trials") OR (DE "Random Assignment") |
| 16 | ((((((TI("randomized controlled trial*") OR AB("randomized controlled trial*")) OR (TI("controlled clinical trial*") OR AB("controlled clinical trial*"))) OR (TI("randomized clinical trial*") OR AB("randomized clinical trial*"))) OR (TI(randomly) OR AB(randomly))) OR (TI(randomized) OR AB(randomized))) OR (TI(trial) OR AB(trial))) OR (TI(placebo) OR AB(placebo)) OR (TI(RCT) OR AB(RCT)) |
| 17 | ((DE "Randomized Controlled Trials") OR (DE "Clinical Trials") OR (DE "Random Assignment")) OR (((((((TI("randomized controlled trial*") OR AB("randomized controlled trial*")) OR (TI("controlled clinical trial*") OR AB("controlled clinical trial*"))) OR (TI("randomized clinical trial*") OR AB("randomized clinical trial*"))) OR (TI(randomly) OR AB(randomly))) OR (TI(randomized) OR AB(randomized))) OR (TI(trial) OR AB(trial))) OR (TI(placebo) OR AB(placebo)) OR (TI(RCT) OR AB(RCT))) |
| 18 | #14 AND #17 |

**Table S7.** Search strategy of ProQuest Dissertations & Theses

| **#** | **Searches** |
| --- | --- |
| 1 | (TI(Universit*) OR AB(Universit*)) OR (TI("college student*") OR AB("college student*")) OR (TI(undergraduate*) OR AB(undergraduate*)) OR (TI(postgraduate*) OR AB(postgraduate*)) OR (TI(student*) OR AB(student*)) |
| 2 | (((((((((((TI(Exercise*) OR AB(Exercise*)) OR (TI("physical activity") OR AB("physical activity"))) OR (TI("physical exercise*") OR AB("physical exercise*"))) OR (TI("aerobic exercise*") OR AB("aerobic exercise*"))) OR (TI("resistance training*") OR AB("resistance training*"))) OR (TI("exercise training*") OR AB("exercise training*"))) OR (TI(sport*) OR AB(sport*))) OR (TI(yoga) OR AB(yoga))) OR (TI("tai chi") OR AB("tai chi"))) OR (TI(qigong) OR AB(qigong))) OR (TI(training) OR AB(training))) |
| 3 | ((((((TI("sleep quality") OR AB("sleep quality")) OR (TI(sleep) OR AB(sleep))) OR (TI(insomnia) OR AB(insomnia))) OR (TI("Pittsburgh Sleep Quality Index") OR AB("Pittsburgh Sleep Quality Index"))) OR (TI(PSQI) OR AB(PSQI)))) |
| 4 | #1 AND #2 AND #3 |
| 5 | ((TI("meta-analysis") OR AB("meta-analysis")) OR (TI("systematic review") OR AB("systematic review"))) |
| 6 | #4 NOT #5 |
| 7 | ((((((TI("randomized controlled trial*") OR AB("randomized controlled trial*")) OR (TI("controlled clinical trial*") OR AB("controlled clinical trial*"))) OR (TI("randomized clinical trial*") OR AB("randomized clinical trial*"))) OR (TI(randomly) OR AB(randomly))) OR (TI(randomized) OR AB(randomized))) OR (TI(trial) OR AB(trial))) OR (TI(placebo) OR AB(placebo)) OR (TI(RCT) OR AB(RCT)) |
| 8 | #6 AND #7 |

**Appendix 3: Risk of bias assessment**

**Figure S3.1:** Overall risk of bias presented as percentage of each risk of bias item across all included studies.

Green = Low risk, Red = High risk, Yellow = Some concerns.

**Appendix 4: Detailed forest plot**

**Figure S4.1.** Forest plot of the effect of exercise on PSQI global score.

**Figure S4.2.** Forest plot of the effect of exercise on sleep latency.

**Figure S4.3.** Forest plot of the effect of exercise on sleep duration.

**Narrative summary: de Vries (2016)**

De Vries et al. assessed sleep duration as self-reported mean nightly sleep hours rather than the PSQI sleep-duration component score. At post-intervention, mean nightly sleep hours were 7.45 ± 0.87 in the exercise group and 7.42 ± 0.90 in the control group, with no statistically significant between-group difference (Group × Time interaction: F = 0.38, p > 0.05). The authors attributed the absence of an effect to a ceiling effect, noting that 82.8% of participants already slept within the recommended 7–9 hours at baseline, leaving limited room for improvement. This finding is consistent with the direction of the primary analysis but cannot be directly pooled with PSQI-based sleep duration estimates due to differences in scale and scoring direction.

**Figure S4.4.** Forest plot of the effect of exercise on sleep efficiency.

**Figure S4.5.** Forest plot of the effect of exercise on sleep disturbances.

**Appendix 5: Leave-one-out sensitivity analysis**

**Figure S5.1.** Leave-one-out sensitivity analysis for the effect of exercise on PSQI global score.

**Figure S5.2.** Leave-one-out sensitivity analysis for the effect of exercise on sleep latency.

**Figure S5.3.** Leave-one-out sensitivity analysis for the effect of exercise on sleep duration.

**Figure S5.4.** Leave-one-out sensitivity analysis for the effect of exercise on sleep efficiency.

**Figure S5.5.** Leave-one-out sensitivity analysis for the effect of exercise on sleep disturbances.

·

**Appendix 6:** **Subgroup analysis by exercise type**

**Figure S6.1.** Subgroup analysis by exercise type for the effect of exercise on PSQI global score.

**Appendix 7:** **Exploratory funnel plots**

**Figure S7.1.** Exploratory funnel plot for the effect of exercise on PSQI global score.

**Figure S7.2.** Exploratory funnel plot for the effect of exercise on sleep latency.

**Figure S7.3.** Exploratory funnel plot for the effect of exercise on sleep duration.

**Figure S7.4.** Exploratory funnel plot for the effect of exercise on sleep efficiency.

**Figure S7.5.** Exploratory funnel plot for the effect of exercise on sleep disturbances.

**Appendix 8:** **Subgroup analyses for PSQI global score**

**Figure S8.1.** Subgroup analysis of the effect of exercise on PSQI global score by intervention duration (≤8 weeks vs. >8 weeks).

**Figure S8.2.** Subgroup analysis of the effect of exercise on PSQI global score by participant sex (All-female vs. Mixed-sex).

**Appendix 9:** **Exploratory Egger's test for publication bias across sleep quality outcomes.**

| **Outcome** | **Number of studies (k)** | **Bias Coefficient** | **Standard Error** | **t-value** | **P-value** | **95% Confidence Interval** |
| --- | --- | --- | --- | --- | --- | --- |
| PSQI global score | 7 | -6.54 | 1.91 | -3.42 | 0.019* | -11.46 to -1.63 |
| Sleep latency | 3 | 34.41 | 27.22 | 1.26 | 0.426 | -311.48 to 380.31 |
| Sleep duration | 3 | 6.33 | 6.34 | 1.00 | 0.501 | -74.26 to 86.91 |
| Sleep efficiency | 3 | -31.53 | NE | NE | NE | NE |
| Sleep disturbances | 3 | -0.92 | 7.97 | -0.12 | 0.927 | -102.25 to 100.40 |

**Note:** NE = Not Estimable. P < 0.05 indicates significant asymmetry in the exploratory funnel plot. For sleep efficiency, test statistics (Standard Error, t-value, P-value, and 95% CI) could not be estimated due to the extremely limited number of studies (k = 3) and insufficient residual variance. Overall, given that the number of included trials across all outcomes is insufficient (k < 10) to ensure adequate statistical power, these exploratory results should be interpreted with extreme caution.

**Appendix 10:** **GRADE assessment**

| Outcome | Risk of bias | Inconsistency | Indirectness | Imprecision | Publication bias | Overall certainty |
| --- | --- | --- | --- | --- | --- | --- |
| PSQI global score | Serious | Serious | Not serious | Not serious | Serious | **Very low** |
| Sleep latency | Serious | Serious | Not serious | Serious | Not assessable | **Very low** |
| Sleep duration | Serious | Serious | Not serious | Not serious | Not assessable | **Very low** |
| Sleep efficiency | Serious | Not serious | Not serious | Not serious | **Not assessable** | **Low** |
| Sleep disturbances | Serious | Serious | Not serious | Serious | Not assessable | **Very low** |

**Risk of bias:** Downgraded by one level (Serious) for all outcomes, as six of the seven included trials were rated as having some concerns in at least one RoB 2 domain, most notably domain D4 (measurement of the outcome), reflecting the inability to blind participants to exercise allocation and the reliance on self-reported sleep instruments that may be susceptible to response bias.

**Inconsistency:** Downgraded by one level (Serious) if there was substantial and unexplained between-study heterogeneity (e.g., I² > 50%) or poor overlap of confidence intervals.

**Indirectness:** Downgraded by one level (Serious) if any dimension of the PICO elements did not directly match the targeted clinical question. Imprecision: Downgraded by one level (Serious) if the total sample size was less than the optimal information size (e.g., n < 400) or if the 95% confidence intervals were wide and crossed the line of no effect. Publication bias: Downgraded by one level (Serious) if visual inspection of the funnel plot showed obvious asymmetry or if Egger's test indicated significant bias (p < 0.10). Rated as "Not assessable" when the number of included trials was insufficient (k < 10) to reliably perform these tests.

**Appendix 11: Sensitivity analysis using change-from-baseline scores**

**11.1 Rationale and methods**

The primary analysis used post-intervention means and standard deviations as the basis for effect size estimation. To evaluate the robustness of these findings in the context of substantial baseline heterogeneity in sleep quality across trials — with baseline PSQI global scores ranging from 3.7 to 9.3 — a pre-specified sensitivity analysis was conducted using change-from-baseline scores as an alternative outcome metric.

As no included trial reported the standard deviation of change scores directly, these were estimated from the reported baseline and post-intervention means and standard deviations using the formula recommended by the Cochrane Handbook for Systematic Reviews of Interventions:

SD_change_= $\sqrt{{SD}_{pre}^{2} + \mathrm{SD}_{\mathrm{post}}^{2} -2 \times r \times\mathrm{SD}_{\mathrm{pre}} \times\mathrm{SD}_{\mathrm{post}}}$

A pre–post correlation coefficient of r = 0.5 was assumed for all trials, consistent with Cochrane Handbook guidance for situations in which individual participant-level correlation data are unavailable. This value represents a conservative and widely adopted assumption in meta-analytic practice. The sole exception was Li (2015), for which change score standard deviations were reported directly in the original publication and were therefore used without estimation.

All sensitivity analyses followed an identical statistical framework to the primary analysis, including random-effects models with DerSimonian–Laird estimation, mean difference (MD) as the summary effect measure, leave-one-out influence analysis, subgroup analysis by exercise modality, funnel plot visualization, and Egger's test for small-study effects. Results for PSQI global score and each of the four PSQI component outcomes are presented in Sections 11.2 through 11.6.

Note that in this appendix, a positive MD reflects greater improvement in the exercise group relative to control (i.e., a larger reduction from baseline), which is the inverse of the sign convention adopted in the primary analysis, where negative values indicated improvement.

**11.2 PSQI global score**

**11.2.1** Forest plot

Note：positive values favor exercise because change scores were calculated as baseline minus post-intervention values.

**11.2.2** Detailed forest plot

**11.2.3** Leave-one-out analysis

**11.2.4** Subgroup analysis by exercise type

**11.2.5** Funnel plot

**11.3 Sleep latency**

**11.3.1** Forest plot

**11.3.2** Detailed forest plot

**11.3.3** Leave-one-out analysis

**11.3.4** Funnel plot

**11.4 Sleep duration**

**11.4.1** Forest plot

**11.4.2** Detailed forest plot

**11.4.3** Leave-one-out analysis

**11.4.4** Funnel plot

**11.5 Sleep efficiency**

**11.5.1** Forest plot

**11.5.2** Detailed forest plot

**11.5.3** Leave-one-out analysis

**11.5.4** Funnel plot

**11.6 Sleep disturbances**

**11.6.1** Forest plot

**11.6.2** Detailed forest plot

**11.6.3** Leave-one-out analysis

**11.6.4** Funnel plot

**11.7 Summary of Egger's Test Results for Change-from-Baseline Sensitivity Analysis**

| **Outcome** | **Number of studies (k)** | **Bias Coefficient** | **Standard Error** | **t-value** | **P-value** | **95% Confidence Interval** |
| --- | --- | --- | --- | --- | --- | --- |
| PSQI global score | 7 | 7.29 | 2.14 | 3.40 | 0.019 | 1.77 to 12.80 |
| Sleep latency | 3 | -11.65 | 26.12 | -0.45 | 0.733 | -343.56 to 320.26 |
| Sleep duration | 3 | -0.38 | 4.22 | -0.09 | 0.943 | -54.05 to 53.28 |
| Sleep efficiency | 3 | -3.52 | 0.30 | -11.53 | 0.055 | -7.39 to 0.36 |
| Sleep disturbances | 3 | 1.15 | 6.92 | 0.17 | 0.895 | -86.75 to 89.04 |

Note: NE = Not Estimable. P < 0.05 indicates significant asymmetry in the exploratory funnel plot. Given that the number of included trials across all outcomes remains insufficient (k < 10) to ensure adequate statistical power for formal publication bias testing, these exploratory results should be interpreted with extreme caution. For any outcome in which test statistics could not be estimated due to extremely limited study numbers and insufficient residual variance, values are reported as NE.

**Appendix 12: Robustness checks for the primary outcome — prediction interval, REML estimation, and Hartung–Knapp correction**

**12.1 PSQI global score**

**12.1.1** DerSimonian–Laird model with 95% prediction interval

**12.1.2** REML estimation with 95% prediction interval

**12.1.3** REML estimation with Hartung–Knapp correction and 95% prediction interval

**12.2 Sleep latency**

**12.2.1** DerSimonian–Laird model with 95% prediction interval

**12.2.2** REML estimation with 95% prediction interval

**12.2.3** REML estimation with Hartung–Knapp correction and 95% prediction interval

**12.3 Sleep duration**

**12.3.1** DerSimonian–Laird model with 95% prediction interval

**12.3.2** REML estimation with 95% prediction interval

**12.3.3** REML estimation with Hartung–Knapp correction and 95% prediction interval

**12.4 Sleep efficiency**

**12.4.1** DerSimonian–Laird model with 95% prediction interval

**12.4.2** REML estimation with 95% prediction interval

**12.4.3** REML estimation with Hartung–Knapp correction and 95% prediction interval

**12.5** **Sleep disturbances**

**12.5.1** DerSimonian–Laird model with 95% prediction interval

**12.5.2** REML estimation with 95% prediction interval

**12.5.3** REML estimation with Hartung–Knapp correction and 95% prediction interval
